# Mendelian randomisation studies of Attention Deficit Hyperactivity Disorder

**DOI:** 10.1002/jcv2.12117

**Published:** 2022-12-07

**Authors:** Lucy Riglin, Evie Stergiakouli

**Affiliations:** ^1^ Division of Psychological Medicine and Clinical Neurosciences and MRC Centre for Neuropsychiatric Genetics and Genomics Cardiff University Cardiff UK; ^2^ Wolfson Centre for Young People's Mental Health Cardiff UK; ^3^ MRC Integrative Epidemiology Unit University of Bristol Bristol UK; ^4^ Population Health Sciences Bristol Medical School University of Bristol Bristol UK

**Keywords:** ADHD, causal inference, mendelian randomisation

## Abstract

**Background:**

Observational studies have found Attention Deficit Hyperactivity Disorder (ADHD) to be associated with an increased risk of adverse outcomes as well as with early risk factors; however it is not clear whether these associations reflect causal effects. Alternatives to traditional observational studies are needed to investigate causality: one such design is Mendelian randomization (MR), which uses genetic variants as instrumental variables for the exposure.

**Methods:**

In this review we summarise findings from approximately 50 studies using MR to examine potentially causal associations with ADHD as either an exposure or outcome.

**Results:**

To‐date, few MR ADHD studies have investigated causal evidence with other neurodevelopmental, mental health and neurodegenerative conditions but those that have suggest a complex relationship with autism, some evidence of a causal effect on depression and limited evidence of a causal effect on neurodegenerative conditions. For substance use, MR studies provide evidence consistent with a causal effect of ADHD on smoking initiation, but findings for other smoking behaviours and cannabis use are less consistent. Studies of physical health suggest bidirectional causal effects with higher body mass index, with stronger effects for childhood obesity, as well as some evidence of causal effects on coronary artery disease and stroke in adults and limited evidence of causal effects on other physical health problems or sleep. Studies suggest bidirectional relationships between ADHD and socio‐economic markers and provide some evidence that low birthweight may be a causal risk factor for ADHD, while bidirectional evidence has been found for some environmental factors. Finally, there is emerging evidence of bidirectional causal links between ADHD genetic liability and biological markers of human metabolism and inflammation.

**Conclusions:**

While MR has advantages over traditional observational designs in addressing causality, we discuss limitations of current ADHD studies and future directions, including the need for larger genome‐wide association studies (and using samples of different ancestries), and for triangulation with different methods.

## Graphical abstract

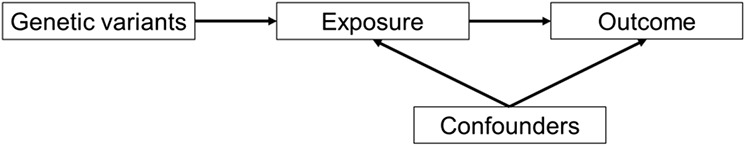

Observational studies have found ADHD to be associated with an increased risk of adverse outcomes as well as with early risk factors; however it is not clear whether these associations reflect causal relationships. In this review we summarise findings from approximately 50 studies using Mendelian randomization (MR) to examine causality with ADHD as either an exposure or outcome.

**Key points**

- Observational studies have found ADHD to be associated with an increased risk of both adverse outcomes and early risk factors: it is not clear whether these reflect causal evidence.
- We summarise findings from approximately 50 studies using MR to examine causality with ADHD as either the exposure or outcome.
- MR studies have examined causal evidence between ADHD and (i) neurodevelopmental, mental health, neurodegenerative conditions and personality, (ii) substance use, (iii) a range of physical health and sleep measures, (iv) cognitive ability test scores and indicators of socio‐economic status, (v) birthweight, gestational age and environmental exposures, and (vi) biological markers.
- MR has advantages over traditional observational designs in examining causal evidence in ADHD, which could have prevention and treatment implications.

Attention Deficit Hyperactivity Disorder (ADHD) is a neurodevelopmental condition that typically onsets in early in development (Diagnostic and Statistical Manual of Mental Disorders, 5th Edition, 2013) and is a common reason for referral to child and adolescent mental health services (Hansen et al., 2021). Observational studies have found ADHD to be associated with an increased risk of adverse outcomes including mental and physical health problems, substance use, lower educational attainment and socio‐economic problems (Angold et al., 1999; Groenman et al., 2017; Instanes et al., 2016; Klein et al., 2012; Loe & Feldman, 2007; Thapar & Cooper, 2016). However, it is not clear whether these associations reflect causal evidence, shared genetic or environmental risk factors (the same factors independently contributing to an increased risk of both ADHD and adverse outcomes) and/or unmeasured confounding and in some cases reverse causation (the outcome of interest causing the exposure of interest). These distinctions are of both theoretical and practical importance as they have different prevention and treatment implications.

In addition to a range of different outcomes, Mendelian randomization (MR) studies have identified several risk factors for ADHD. ADHD is highly heritable – twin studies estimating 70%–80% heritability and limited evidence for shared familial environmental contributions (Brikell et al., 2021) – and many genetic and environmental factors likely contribute to onset to varying degrees for most individuals with ADHD (Thapar & Cooper, 2016). Observational studies have found prenatal, perinatal and early‐environmental factors to be associated with ADHD (Thapar et al., 2013). Longitudinal and (quasi‐)experimental designs suggest that some of these associations are unlikely to be causal (Thapar & Cooper, 2016). For example, longitudinal studies, adoption studies and treatment trials suggest that negative mother‐child relationships are likely outcomes rather than causes of ADHD (Harold et al., 2013; Lifford et al., 2009; Schachar et al., 1987) and quasi‐experimental studies, such as using a cohort of patients undergoing in vitro fertilization, suggest that associations between prenatal smoking and ADHD are likely due to unmeasured confounding (Rice et al., 2018). Again, disentangling the nature of the association between potential risk factors and ADHD has theoretical, prevention and treatment implications.

Thus, observational studies have found ADHD is associated with a range of both exposures and outcomes, but it is not clear if these associations are causal. Alternatives to traditional observational studies are needed to investigate potentially causal evidence while minimising bias due to unmeasured confounding and reverse causation. One such alternative is MR, which uses genetic variants identified from genome‐wide association studies (GWAS) as instrumental variables (proxies) for the exposure: as shown in Figure 1, MR is based on the premise that while observational associations between exposures and outcomes can be due to confounders, if genetic variants are not associated with confounders, these can be used as unconfounded proxies for the exposure. As shown in Figure 2, primary assumptions of MR (Davey Smith & Hemani, 2014) are that these genetic proxies should be (i) reliably associated with the exposure of interest (relevance assumption): for example, genetic variants showing genome‐wide significance in a large GWAS. (ii) independent of confounders of the exposure‐outcome association (independence assumption): relevant confounders should not be associated with the genetic variants. (iii) associated with the outcome of interest only via the exposure (exclusion restriction assumption): genetic variants should not show independent associations with the outcome (i.e. horizontal pleiotropy, see Table 1). Since genetic variants are randomly assigned at conception, exposure‐outcome associations (e.g. between ADHD genetic liability and outcomes) will suffer from minimal confounding and will be somewhat analogous to a randomised control trial (e.g. in which the intervention increases the likelihood of ADHD) (Davey Smith & Hemani, 2014). Key MR definitions are given in Table 1.

**FIGURE 1:**
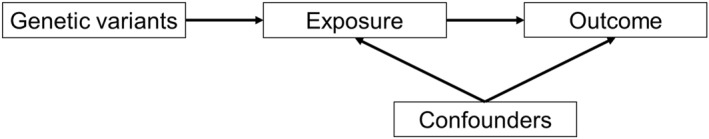
Mendelian randomization (MR)

**FIGURE 2:**
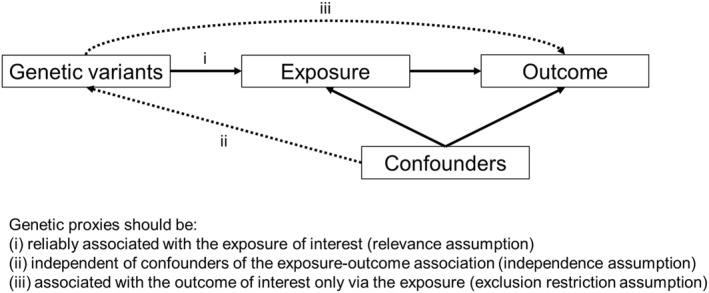
Mendelian randomization (MR) primary assumptions

**TABLE 1:** Mendelian randomization (MR) definitions

Term	Definition	Relevant analysis
Causal effect	Genetic liability for the exposure is causing the outcome.	The slope of an MR regression examining the association between genetic liability to the exposure and the outcome.
Bidirectional relationship	Evidence consistent with a causal effect of the exposure on the outcome and a causal effect of the outcome on the exposure (the latter suggesting reverse causation).	Two MR analyses: one investigating the effect of genetic liability to the exposure on the outcome and one investigating the effect of genetic liability to the outcome on the exposure.
Horizontal pleiotropy	Genetic variants for the exposure are associated with the outcome via other routes than the exposure (genetic liability to the exposure is associated with the outcome in the absence of the exposure).	The intercept of an MR regression examining the association between genetic liability to the exposure and the outcome.
Heterogeneity	Genetic variants show variability in the estimated exposure‐outcome association.	Dependent on the type of MR analysis.

In this paper we will summarise findings from studies using MR to examine potentially causal evidence with ADHD as either an exposure or outcome. We focus on 2‐sample MR studies, in which data on the exposure and outcome of interest are identified from summary statistics of two different GWAS (one for the exposure and one for the outcome), although some alternative designs are briefly covered. Our search strategy for identifying MR studies of ADHD is outlined in the Supporting Information.

## NEURODEVELOPMENTAL, MENTAL HEALTH, NEURODEGENERATIVE CONDITIONS AND PERSONALITY

Despite substantial phenotypic overlap, few MR studies have investigated causal evidence between ADHD and other conditions involving the brain, mainly due to the challenges of identifying causal effects with genetic instruments that display extensive pleiotropic effects across different conditions. Two studies of ADHD and autism spectrum disorder (ASD) suggested a complex relationship, with evidence of a causal effect of genetic liability to ASD on ADHD and of genetic liability to ADHD on ASD, with effects in both directions showing substantial heterogeneity (Baranova et al., 2022; Peyre et al., 2021) and evidence of horizontal pleiotropy (Peyre et al., 2021), likely reflecting the strong shared heritability between these conditions. This is one example of the complexities of applying MR in the context of conditions that show strong genetic overlap: identifying genetic proxies which are associated with the outcome of interest only via the exposure may be particularly challenging in such situations.

Two MR studies of ADHD and depression found evidence of a causal effect of ADHD genetic liability on major depression, but effect estimates were weaker for an effect on “broad depression” (self‐reported help‐seeking behaviour for nerves, anxiety, tension or depression) (Riglin et al., 2021; Soler Artigas et al., 2022), for which there was evidence of heterogeneity and horizontal pleiotropy (Riglin et al., 2021). Analyses in the reverse direction did not find strong evidence of an effect of major depression genetic liability on ADHD (Riglin et al., 2021; Soler Artigas et al., 2022), with evidence of heterogeneity (Riglin et al., 2021) (although these analyses were limited by the small number of SNPs available for major depression). One of these studies found a causal effect of genetic liability to broad depression on ADHD, with evidence of heterogeneity but little horizontal pleiotropy (Riglin et al., 2021) while the other did not find strong evidence in this direction (Soler Artigas et al., 2022). These studies therefore provide evidence for a causal effect of ADHD on major depression, but suggest that broad depression may impact ADHD: given that ADHD precedes depression, this may suggest parental effects or the influence of early temperament which overlaps with several mental health traits (Riglin et al., 2021). Another MR study found ADHD genetic liability to also increase risk of self‐harm, although multivariable analyses suggested that this was not independent of the causal effect of genetic liability to depression (Lim et al., 2020). One MR study investigating associations with bipolar disorder did not find evidence of causal effects in either direction (Soler Artigas et al., 2022).

Three MR studies have investigated causal evidence between ADHD and measures of personality. Two studies investigated neuroticism – a personality trait characterised by negative emotionality – and found some evidence of a causal effect of neuroticism genetic liability on ADHD but not strong evidence of an effect in the other direction (Soler Artigas et al., 2022; Zhang et al., 2021), although for one study this effect did not meet their study sensitivity criteria (Soler Artigas et al., 2022). One of these studies also examined several traits relating to personality and specific mood variables or help seeking behaviour (Soler Artigas et al., 2022): ADHD genetic liability had a causal effect on being able to confide, feeling lonely and the frequency of both depressed mood and unenthusiasm/disinterest in the last 2 weeks with evidence of bidirectional effects for all but feeling lonely (Soler Artigas et al., 2022). However, only causal effects from ADHD genetic liability to unenthusiasm/disinterest and from genetic liability to being able to confide on ADHD met sensitivity criteria. As was previously observed for broad measures of depression (Riglin et al., 2021), genetic liability to eight mood measures had causal effects on ADHD although only liability to frequency of tiredness/lethargy in the last 2 weeks met sensitivity criteria (Soler Artigas et al., 2022). Finally, a different study examining causal evidence with the personality trait extraversion found evidence of a causal effect of genetic liability to extraversion on ADHD (Baranova et al., 2022).

Only a handful of studies have investigated causal effects of ADHD and neurodegenerative conditions. Longitudinal studies investigating the effect of childhood diseases, such as ADHD, on neurodegenerative conditions would require a very long follow‐up period and would suffer by large amounts of attrition. Mendelian randomization studies are well‐suited in studying causal effects on outcomes that happen a long time after the exposure. A study examining causal evidence with Parkinson's disease did not find strong evidence of a causal effect of ADHD genetic liability on Parkinson's disease (Li et al., 2020). This study did find some evidence of a causal effect in the reverse direction, which was not accounted for by body mass index (BMI) or smoking, however the authors suggest this could be driven by unmeasured confounders (Li et al., 2020). A more recent study using a smaller Parkinson's disease GWAS did not find strong evidence of a causal effect on ADHD (Kim et al., 2021). Finally, according to a recent bidirectional MR study, there was very little evidence of a causal effect of genetic liability to ADHD on Alzheimer's disease (the same was true on the other direction) (Pagoni et al., 2020): this was also true when the effects of educational attainment and intelligence quotient were taken into account.

Thus, MR studies of ADHD suggest a complex relationship with ASD, some evidence of a causal effect on depression and limited evidence of a causal effect on neurodegenerative conditions. There is also some evidence that negative emotionality may have a causal effect on ADHD.

## SUBSTANCE USE

### Smoking

A number of studies have used MR to examine causal evidence between ADHD and various smoking‐related traits. Four studies examining smoking initiation have found evidence of causal effects of genetic liability to ADHD on smoking (Fluharty et al., 2018; Jang et al., 2022; Soler Artigas et al., 2022; Treur et al., 2021; Vilar‐Ribó et al., 2021) with evidence of heterogeneity (Jang et al., 2022; Treur et al., 2021; Vilar‐Ribó et al., 2021) or horizontal pleiotropy (Fluharty et al., 2018). Three of these studies examined effects in the opposite direction and also found evidence of causal effects of genetic liability to smoking initiation on ADHD with limited heterogeneity or horizontal pleiotropy (Treur et al., 2021; Vilar‐Ribó et al., 2021), although one result did not meet study sensitivity criteria (Soler Artigas et al., 2022) and a fourth study did not find strong evidence in this direction (Jang et al., 2022). Again, as ADHD precedes smoking, these positive findings may reflect unmeasured confounding or parental effects or pleiotropy.

Findings of studies examining other smoking phenotypes are less consistent. For example, for age of smoking initiation, one study did not find clear evidence of a causal effect of ADHD genetic liability (with little evidence of heterogeneity or horizontal pleiotropy) (Fluharty et al., 2018), whereas two more recent studies using a larger smoking GWAS and limiting the ADHD GWAS to European samples did find evidence of a causal effect (with little evidence of horizontal pleiotropy) (Jang et al., 2022; Vilar‐Ribó et al., 2021) with one finding evidence of heterogeneity (Vilar‐Ribó et al., 2021). For number of cigarettes per day smoked, two studies have found evidence of a causal effect of ADHD genetic liability on cigarettes per day (Treur et al., 2021; Vilar‐Ribó et al., 2021) but another with additional smoking GWAS samples found weaker evidence (Jang et al., 2022).

For smoking cessation, two studies did not find evidence of a causal effect of ADHD (Jang et al., 2022; Vilar‐Ribó et al., 2021) while another two did (Soler Artigas et al., 2022; Treur et al., 2021): two of these reaching different conclusions used the same smoking GWAS, but the one with a null result excluded overlapping samples from the ADHD GWAS (Vilar‐Ribó et al., 2021). One of these also found evidence in the opposite direction: of genetic liability to past smoking on ADHD (Soler Artigas et al., 2022). The later study investigated several smoking phenotypes and found evidence (passing sensitivity criteria) for an effect of ADHD genetic liability to both current smoking and pack years adult smoking as proportion of life span exposed to smoking (Soler Artigas et al., 2022). Finally, one study of nicotine dependence did not find strong evidence of a causal effect of ADHD genetic liability (Vink et al., 2021).

Thus, MR studies provide evidence consistent with a causal effect of ADHD on smoking initiation, but findings for other smoking behaviours are less consistent.

### Cannabis use

Studies investigating cannabis use have generally found evidence of a causal effect of ADHD genetic liability, including for cannabis use initiation (Treur et al., 2021). Studies examining lifetime cannabis use have found mixed results: two found evidence of a causal effect on lifetime cannabis use (Soler Artigas et al., 2020; Vilar‐Ribó et al., 2021) while another did not (Jang et al., 2022). One of the studies which found evidence of a causal effect utilised an older cannabis GWAS with fewer samples (Soler Artigas et al., 2020), but two studies with conflicting findings used the same cannabis GWAS and the same *p*‐value threshold for inclusion of SNPs (*p* < 5 × 10^−08^) (Jang et al., 2022; Vilar‐Ribó et al., 2021), although the study with null findings included fewer SNPs. Another difference is that the studies which found causal evidence excluded overlapping samples from the ADHD GWAS used (Soler Artigas et al., 2020; Vilar‐Ribó et al., 2021) while the study with null findings did not (Jang et al., 2022). Where this was tested, the cannabis studies found limited evidence of heterogeneity or horizontal pleiotropy for causal effect of ADHD (Jang et al., 2022; Soler Artigas et al., 2020; Treur et al., 2021; Vilar‐Ribó et al., 2021). Examining the reverse direction, one of the studies using the larger lifetime cannabis use GWAS found evidence of a causal effect of genetic liability to cannabis on ADHD (Vilar‐Ribó et al., 2021) whereas the other studies did not (Jang et al., 2022; Soler Artigas et al., 2020; Treur et al., 2021). Thus, studies have found evidence of a causal effect of ADHD genetic liability on cannabis use, although this is not consistent across all studies, with weaker evidence of causal effects in the reverse direction.

### Other substances: Alcohol, illicit drugs and coffee

Studies have provided mixed evidence of a causal effect of genetic liability to ADHD on alcohol use. Two studies using similar GWAS have not found strong evidence of an effect on alcoholic drinks per week, with evidence of heterogeneity but limited evidence of horizontal pleiotropy (Jang et al., 2022; Treur et al., 2021) while another (using a smaller alcohol GWAS) found evidence of effects on alcohol intake frequency and intake versus 10 years previously (Soler Artigas et al., 2022); strong evidence was also not found for effects of genetic liability to alcoholic drinks per day on ADHD (Jang et al., 2022; Treur et al., 2021) although these was for genetic liability to alcohol intake frequency and intake versus 10 years previously (Soler Artigas et al., 2022).

One study did not find evidence of a causal effect in either direction for alcohol problems (Treur et al., 2021). Finally, one study of alcohol dependence did not find strong evidence of a causal effect of ADHD genetic liability on alcohol dependence (Vilar‐Ribó et al., 2021) with weak evidence found by another, using a larger alcohol GWAS (Treur et al., 2021); neither found strong evidence of effects in the reverse direction.

One of these substance use studies also examined cocaine dependence and addiction to illicit drugs (Vilar‐Ribó et al., 2021): the authors did not find strong evidence of a causal effect of ADHD genetic liability, although the substance use GWAS used for these were smaller than others examined in the study, which may have limited power to detect effects. Finally, one study which examined coffee consumption did not find evidence of causal effects of ADHD in either direction (Treur et al., 2021).

Mendelian randomization studies have therefore not provided evidence to support a causal effect of ADHD on substance use, although this may in part be due to limited sample sizes.

## PHYSICAL HEALTH AND SLEEP

Observational studies have shown wide‐spread associations of ADHD with adverse physical health outcomes. One MR study investigated the effect of genetic liability to ADHD on a range of physical health outcomes and reported causal effects on childhood obesity, coronary artery disease, smoking and (in the presence of horizontal pleiotropy) inflammatory bowel disease (Leppert et al., 2021). Using multivariable MR they also identified evidence of the effect of ADHD genetic liability on coronary artery disease being mediated by childhood obesity. There was also bidirectional causal evidence between ADHD and childhood obesity. The authors did not identify causal evidence for ADHD genetic liability on BMI, myocardial infarction, hypertension, systolic blood pressure, type 2 diabetes mellitus, migraine, epilepsy, autoimmune and allergic diseases and lung cancer.

Another recent MR study examined causal links between ADHD genetic liability and 124 traits including 20 anthropometric traits, lifestyle and environment, longevity, neurological, psychiatric and mental health or personality and psychosocial factors available in the MR‐Base database (Soler Artigas et al., 2022). They reported limited evidence of a causal effect of ADHD genetic liability on the anthropometric traits and evidence of an effect of the genetic liability of eight traits (arm, leg, whole body and trunk fat‐free mass, arm and trunk predicted mass, whole body water mass and weight) on ADHD. However, they identified causal effects of ADHD genetic liability on all measures of longevity examined. They also reported some evidence of ADHD genetic liability on activity measures.

Causal effects of genetic liability to ADHD on higher BMI and obesity have been investigated by further four MR studies on top of the previously discussed (Leppert et al., 2021; Soler Artigas et al., 2022). One study reported causal effects of ADHD genetic liability on higher BMI, waist circumference, waist‐hip‐ratio and BMI‐adjusted waist‐hip‐ratio, with limited evidence for body fat percentage and basal metabolic rate (Karhunen et al., 2021). There was also evidence of bidirectional effects of genetic liability to BMI, waist circumference, body fat percentage and basal metabolic rate on increased ADHD risk. Another study using generalized summary data‐based Mendelian randomization identified some evidence of a causal effect of ADHD genetic liability on higher BMI, fat mass or fat‐free mass but limited evidence on body fat percentage (Hübel et al., 2019). Genetic liability to increased fat mass, fat‐free mass and body fat percentage also had a causal effect on ADHD. Causal effects of genetic liability to ADHD on higher BMI were further reported by Liu et al. (2021) when using data from the largest GWAS meta‐analysis of BMI available (including 806,834 participants from GIANT consortium and UK Biobank) with little evidence of horizontal pleiotropy. They also found a causal effect of higher BMI genetic liability on ADHD. The effect was attenuated but evidence of causal effects still remained when controlling for education. Finally, a bidirectional MR study of genetic liability to ADHD on increased childhood and adult BMI found causal evidence in both directions (Martins‐Silva et al., 2019).

Regarding other physical health outcomes, MR studies on ADHD genetic liability and asthma have reported mixed results with one study failing to find evidence (Leppert et al., 2021) while a second MR study, using data from a larger GWAS of asthma, found causal evidence of genetic liability to ADHD on increased risk of asthma (Zhaozhong et al., 2019). None of the studies identified causal evidence on the opposite direction (genetic liability to asthma on ADHD). Genetic liability to ADHD has been found to be causally linked to higher risk for ischemic stroke and large‐artery atherosclerotic stroke with multivariable MR suggesting that some of the effect of ADHD genetic liability on stroke could be mediated through coronary artery disease (Du et al., 2022). In a bidirectional MR study of genetic liability to ADHD and mouth ulcers no causal evidence was reported on either direction (Wang et al., 2020). The same was also true for a different bidirectional MR study of genetic liability to ADHD and intracranial aneurysms (Peng et al., 2021). Finally, genetic liability to ADHD was investigated for causal effects on the severity of COVID‐19 in a MR framework with strong causal evidence of ADHD genetic liability increasing risk of hospitalization due to COVID‐19 (Liu, Tan, et al., 2021).

Four MR studies have investigated causal effects between ADHD and sleep‐related phenotypes. Mendelian randomization in this context can be particularly valuable in disentangling the effects of ADHD and medications for ADHD on sleep. The first study found limited evidence of bidirectional causal effects of genetic liability to ADHD and insomnia (Gao et al., 2019). However, Sun et al. (2022) identified strong evidence of genetic liability to insomnia being causally linked to ADHD. Bidirectional analysis showed limited evidence of ADHD genetic liability being causal to other sleep‐related phenotypes. A third study reported causal effects of ADHD genetic liability on longer sleep duration and chronotype but not other sleep‐related phenotypes including insomnia (Carpena et al., 2021). There was also some causal evidence of sleep‐related phenotypes on ADHD (Carpena et al., 2021). Finally, Soler Artigas et al. (2022) investigated five sleep traits (daytime dozing, nap during day, sleep duration, insomnia, snoring) on ADHD and did not find strong evidence of a causal effects in either direction.

In conclusion, there is evidence of bidirectional causal effects between increased genetic risk for ADHD and higher BMI, with stronger effects for childhood obesity. There is also evidence that ADHD genetic liability could have causal effects on coronary artery disease and stroke in adults. Some of these causal effects could be linked as has been indicated by multivariable MR (for example, the effect of ADHD genetic liability on coronary artery disease could be mediated by childhood obesity). At the moment, there is limited evidence of ADHD genetic liability causing other physical health problems (apart from increasing risk of hospitalization due to COVID‐19) or affecting sleep.

## COGNITIVE ABILITY TEST SCORES AND INDICATORS OF SOCIO‐ECONOMIC STATUS

Three MR studies investigating ADHD and cognitive ability test scores using the same cognitive ability GWAS have found evidence of a causal effect of genetic liability to higher cognitive ability test scores on lower risk of ADHD, with little evidence of horizontal pleiotropy (Michaëlsson et al., 2022; Rao et al., 2022; Savage et al., 2018) although a fourth did not (Soler Artigas et al., 2022). In the reverse direction, one of these studies also found evidence of a causal effect of ADHD genetic liability on lower cognitive ability test scores, although of a smaller magnitude (Savage et al., 2018) while the others did not find strong evidence in this direction (Michaëlsson et al., 2022; Soler Artigas et al., 2022). One of these studies examined causal evidence with four additional measures of cognitive function and reported evidence of a causal effect of ADHD on cognitive performance, weaker evidence for fluid intelligence and little evidence for two cognitive tests assessing reaction time and pairs matching. Little evidence was also found for effects of any of these on ADHD (Soler Artigas et al., 2022).

Two of the studies investigating cognitive ability (Michaëlsson et al., 2022; Soler Artigas et al., 2022) along with another (Dardani et al., 2021) also examined causal evidence with educational attainment, all using the same GWAS. All found evidence of causal effects of ADHD genetic liability on lower educational attainment and of educational attainment genetic liability on lower risk of ADHD with limited evidence of horizontal pleiotropy (Dardani et al., 2021; Michaëlsson et al., 2022; Soler Artigas et al., 2022). Moreover, multivariable analyses suggested that effects in both directions were independent of cognitive ability (Dardani et al., 2021; Michaëlsson et al., 2022). Bidirectional causal effects were also found for age of completion of full time education (Soler Artigas et al., 2022). One of these studies examined causality with employment (jobs involving manual or physical work; job involved mainly walking or standing) and did not find strong evidence of effects in either direction (Soler Artigas et al., 2022).

Michaëlsson et al. (2022) also found evidence of bidirectional causal effects between ADHD and both lower household income and higher Townsend deprivation index, with evidence of horizontal pleiotropy in the effects of genetic liability to household income on ADHD. Causal effects between genetic liability to lower income and higher deprivation index on ADHD were both independent of cognitive ability. Soler Artigas et al. (2022) also found evidence of bidirectional effects for lower household income and higher Townsend deprivation index although effects of genetic liability to income on ADHD did not pass sensitivity analyses. The same study also found evidence of a causal effect of ADHD genetic liability on number of full sisters, with weaker evidence for number of brothers and number of vehicles in household and did not find strong evidence for causal effects on home location, length at current address or number of people in household (Soler Artigas et al., 2022).

Thus, current MR studies suggest bidirectional relationships between socio‐economic markers and ADHD: this could reflect an increased likelihood for ADHD symptoms being impairing in the absence of sufficient or additional resources, decline in family income for those with a child with ADHD and that ADHD can lead to barriers in attaining higher education and income.

## BIRTHWEIGHT, GESTATIONAL AGE AND ENVIRONMENTAL EXPOSURES

Three MR studies of birthweight and ADHD have reported inconsistent findings: two did not find strong evidence of a causal effect of low birthweight on ADHD (Arafat & Minică, 2018; Soler Artigas et al., 2022) while another, using a larger birthweight GWAS did find evidence of an effect (Orri et al., 2021) (both finding evidence of heterogeneity). There is therefore some evidence consistent with a causal effect of low birthweight on ADHD, although studies have yet to examine the extent to which the effect of birthweight may be capturing other underlying foetal problems. One study examining causal effects of maternal smoking around birth found initial evidence of bidirectional effects but sensitivity analyses suggested a causal effect of ADHD genetic liability on maternal smoking rather than the other direction (Soler Artigas et al., 2022). The same study did not find strong evidence in either direction for being breastfed as a baby or place of birth in UK (Soler Artigas et al., 2022). Another study did not find strong evidence of a causal effect of genetic liability to gestational duration on ADHD (Yao et al., 2022).

One of these studies investigating multiple environmental factors also found evidence of a causal effect of ADHD genetic liability on two measures of air pollution (nitrogen dioxide and particle matter) and two measures of sexual behaviour (lifetime number of sexual partners and age of first sexual intercourse) with bidirectional effect for the latter (Soler Artigas et al., 2022).

One study examining childhood maltreatment suggested a bidirectional relationship, with evidence of a causal effect of genetic liability to ADHD on childhood maltreatment and a causal effect of genetic liability to childhood maltreatment on ADHD (Warrier et al., 2021). The authors suggested that this could be due to gene‐environment correlation, either whereby parents of children with ADHD (who are more likely to have ADHD traits) may be more likely to maltreat their children (passive gene‐environment correlation) or whereby genetic liability to ADHD is associated with difficulties that increase risk of maltreatment (active gene‐environment), as well supporting a causal explanation (Warrier et al., 2021).

Finally, of two studies examining computerised device use, one found evidence of a bidirectional relationship, such that there was evidence of a causal effect of genetic liability to ADHD on mobile phone use and a causal effect of genetic liability to mobile phone use on ADHD (Wendt et al., 2019) while another found weak evidence for an effect of ADHD genetic liability to playing computer games and of bidirectional effects for mobile phone use with stronger evidence for an effect of genetic liability to both length of mobile phone use and time spent watching TV (Soler Artigas et al., 2022).

Thus MR studies provide some evidence low birthweight may be a causal risk factor for ADHD, while bidirectional associations have been found for some environmental factors.

## BIOLOGICAL MARKERS

Five MR studies have examined causal evidence between biological markers, usually measured in blood, and ADHD. Three of them integrated large ‘omics’ datasets (metabolomics, proteomics and cytokines) with genomics to investigate causal roles of blood biomarkers on ADHD.

One 2‐sample MR study integrated metabolomic and genomics and used data from a GWAS of 486 metabolites (traits of the human metabolism measured in plasma; Shin et al., 2014) to examine causal effects on five psychiatric disorders, including ADHD (Yang et al., 2020): 27 metabolites showed evidence of association with ADHD, with one of them (1‐docosahexaenoylglycerophosphocholine) passing Bonferroni correction as determined in the study protocol. There was very limited evidence of pleiotropic effects. Using all the metabolites that showed evidence of causality, the authors performed metabolic pathway analysis with several pathways found to be associated with ADHD.

In another 2‐sample MR (Yang et al., 2022), the same group used a GWAS of 2994 plasma proteins (Sun et al., 2018) to examine their potential causal role on five psychiatric disorders, including ADHD. Genetically predicted levels of beta‐mannosidase (MANBA) decreased risk of ADHD with no evidence of pleiotropic effects despite the finding being mainly driven by a single genetic instrument.

A third 2‐sample MR study (Chen et al., 2022) used information from a large GWAS of systemic inflammatory regulators (cytokines) (Ahola‐Olli et al., 2017) to test bidirectionally for causality between 41 cytokines and psychiatric disorders, including ADHD. There was evidence for two genetically determined cytokines (beta nerve growth factor and stem cell factor) having a causal role on ADHD. In addition, the same study identified a potential causal role for genetic liability to ADHD and two cytokines (interleukin7 and tumour necrosis factor–alpha). Results did not pass multiple testing correction in all cases. However, cytokines are highly correlated so adjusting for multiple testing might be overly conservative in this case. Sensitivity analyses adjusting for potential pleiotropic effects showed the same pattern of results.

Genetically predicted urate levels (linked to gout) have been tested for causal effects on psychiatric disorders, including ADHD, with the MR study not identifying any potential causal effects (Zhao et al., 2021). The same was also true for genetically predicted 25(OH)vitamin D levels (Libuda et al., 2021). The bidirectional 2‐sample MR of genetically predicted 25(OH)vitamin D levels on ADHD reported no potential causal effects on either direction.

Thus, there is emerging evidence of bidirectional causal links between ADHD genetic liability and biological markers of human metabolism and inflammation.

## OTHER MENDELIAN RANDOMISATION DESIGNS

In this review we focussed on ADHD studies using 2‐sample MR, in which summary statistics from different genetic studies are used to assess causal evidence. However, a range of MR designs exist, utilising different sources of data. For example, individual level data MR can be used when the SNPs, exposure phenotype and outcome phenotype data are all measured within the same sample, and one‐sample MR using summary data can be conducted when the same sample is used to estimate the SNP‐exposure and SNP‐outcome associations (Hemani et al.,  2018). Other related approaches include (a) calculating polygenic scores as indicators of genetic liability to ADHD (Wray et al., 2014) for use in subsequent analyses (this approach requires individual participant genotype data and makes it difficult to test for pleiotropic effects), (b) MR phenome‐wide association study (MR‐pheWAS) to perform hypothesis‐free analyses investigating associations between genetic liability to ADHD and a large number of phenotypes (Millard et al., 2015), and (c) using summary data‐based MR to integrate summary‐level GWAS data with other omic data to investigate whether the effects of ADHD genetic variants are mediated by expression or methylation levels (Zhihong et al., 2016). Attention Deficit Hyperactivity Disorder studies using these and other MR‐based designs are reviewed in the Supporting Information.

## THEORETICAL, PREVENTION AND TREATMENT IMPLICATIONS

As MR provides insight into potentially causal effects (given certain assumptions), ADHD MR study findings – whether ADHD is the exposure or outcome – have theoretical, prevention and treatment implications. Where study findings are consistent with a causal effect of ADHD on outcomes, such as have been observed for depression and smoking initiation, these suggest that effective treatment of ADHD may reduce the risk of these outcomes, although triangulation of findings is needed (see below). Multivariable MR can provide indications of potential causal pathways of these relationships, for example, findings implicating childhood obesity as a mediator of the causal link between ADHD and coronary artery disease (Leppert et al., 2021) suggest that interventions aimed at reducing the risk of this outcome in those with ADHD would need to begin early in childhood. ADHD MR findings of bidirectional relationships, such as that observed for BMI and socio‐economic markers, highlight the complexity of these relationships. Again, effective treatment of ADHD may help reduce these outcomes, but there could also be dynastic effects such that parental BMI/socio‐economic factors could increase risk of ADHD in the offspring. Where MR findings are inconsistent with a causal effect of ADHD (again given MR assumptions, as well as sufficiently powered analyses), this suggests alternative explanations for observational associations, such as shared risk factors or unmeasured confounding. This suggests that effective treatment of ADHD is unlikely to reduce risk of these outcomes (provided other designs suggest the same) and that research into other causal factors and/or using other designs is needed to understand observed associations.

Where MR studies have investigated ADHD as the outcome, findings consistent with a casual effect on ADHD, such as have been observed for low birthweight, suggest that targeting these exposures in those at risk of ADHD may reduce risk of ADHD. However, it is not clear what causal effects of phenotypes such as low birth weight capture, maybe other underlying foetal problems. Finally, emerging evidence of bidirectional causal links between ADHD genetic liability and biological markers of human metabolism and inflammation may provide clues as to the biological underpinnings of ADHD, which could help refine treatment options.

## FUTURE DIRECTIONS FOR MENDELIAN RANDOMISATION STUDIES OF ATTENTION DEFICIT HYPERACTIVITY DISORDER

While MR studies have advantages over traditional observational designs in minimising unmeasured confounding and reverse causation, this is based on MR assumptions (Davey Smith & Hemani, 2014) (e.g. Figure 2) which may not be met, especially for neurodevelopmental and mental health phenotypes where extensive pleiotropic effects are well documented. Indeed, the main source of bias for MR studies is horizontal pleiotropy (Lawlor et al.,  2016); although different methods have been developed to address this, these also have limitations (Hemani et al.,  2018). Other biases include weak instrument bias, which is minimised by the large GWAS available for ADHD (Demontis et al., 2019) but is likely problematic for smaller exposure/outcome GWAS. Thus, to further our understanding of causal relationships with ADHD using MR, further research addressing these issues is needed, including larger, more powerful GWAS to detect genetic variants that are reliably associated with the exposure of interest and to limit horizontal pleiotropy. More research is also needed into both the impact of incomplete/bias GWAS samples on MR studies of ADHD (e.g. see Taylor et al., 2018).

Population stratification is another source of bias in MR: many of the studies we reviewed restricted the ADHD GWAS used to European origin samples (which comprise the majority of samples included in the largest ADHD GWAS; Demontis et al., 2019), but this was not the case for all. 2‐sample MR assumes that both GWAS samples come from the same underlying population, which means that European samples for the exposure/outcome are needed if a European ADHD GWAS is used. A serious limitation of current MR ADHD findings is therefore that findings may only apply to European origin populations and may not generalise to others: future ADHD (and other) GWAS using samples of different ancestries are greatly needed to be able to examine generalisability. Additional potential limitations of MR have been discussed elsewhere by (e.g. Davey Smith & Hemani, 2014).

Finally, triangulation between MR findings and those of other methods is required as different designs will have different sources of bias (Lawlor et al.,  2016); alternative designs including using natural experiments and animal models to study causal evidence have been reviewed elsewhere (e.g. Thapar & Rutter, 2015). Converging evidence from different designs is important to strengthen causal inference for neurodevelopmental conditions (Larsson, 2021).

## CONCLUSIONS

In conclusion, to‐date approximately 50 studies have been published examining potentially causal evidence with ADHD as either the exposure or outcome. Few MR studies investigated causal effects between ADHD and other conditions involving the brain, but those that have suggest a complex relationship with ASD, some evidence of a causal effect on depression and limited evidence of a causal effect on neurodegenerative conditions. Mendelian randomization studies of substance use provide evidence consistent with a causal effect of ADHD on smoking initiation, but findings for other smoking behaviours and cannabis use are less consistent. Several studies have examined causal evidence with different aspects of physical health: these suggest bidirectional causal effects with higher BMI, with stronger effects for childhood obesity, as well as some evidence of causal effects on coronary artery disease and stroke in adults, but limited evidence of causal effects on other physical health problems or sleep. Studies suggest bidirectional relationships between ADHD and socio‐economic markers, which could reflect an increased likelihood for ADHD symptoms being impairing in the absence of sufficient or additional resources and that ADHD can lead to barriers in attaining higher education and income. Studies have also provided some evidence that low birthweight may be a causal risk factor for ADHD, while bidirectional effects have been found for some environmental factors. Finally, there is emerging evidence of bidirectional causal links between ADHD genetic liability and biological markers of human metabolism and inflammation. While MR has advantages over traditional observational designs in addressing causality it is not without limitations and triangulation with different methods is needed to increase confidence in causal association.

## AUTHOR CONTRIBUTIONS

**Lucy Riglin:** Data curation, Investigation, Writing – original draft. **Evie Stergiakouli:** Investigation, Writing – original draft.

## CONFLICT OF INTEREST

The authors have declared that they have no competing or potential conflicts of interest.

## ETHICAL CONSIDERATIONS

No ethics approval was required for this article as no new data were created or analyzed in this study.

## Supporting information

Supplementary Material

## Data Availability

Data sharing is not applicable to this article as no new data were created or analyzed in this study.
