# Supplementary material for: Mendelian randomisation studies of Attention Deficit Hyperactivity Disorder
Source: JCPP Adv. 2022 Dec 7;2(4):e12117. doi: 10.1002/jcv2.12117 (PMC10242846; doi:10.1002/jcv2.12117)
Supplement: Supplementary file 1 — Supplementary Material [file JCV2-2-e12117-s001.docx]

**Supporting Information**

**Search strategy**

We searched PubMed on 29^th^ June 2022 for “(“ADHD” OR “attention deficit hyperactivity disorder” OR “hyperkinetic”) AND (“mendelian random*”)” in any field with no additional restrictions: 52 articles were identified. Summaries of the studies identified are presented in Supplementary Tables 1-6.

**Other Mendelian randomisation (MR) designs**

***Polygenic scores and PheWAS***

Where individual participant genotype data are available, they can also be used to generate polygenic scores (PGS): the sum of the count of risk alleles (identified in the GWAS), weighted by the effect size (Wray et al. 2014). Given the polygenic nature of ADHD, many studies generate ADHD PGS including SNPs associated with ADHD at varying level of evidence, including those that do not pass the genome-wide significance threshold for association. ADHD PGS are thus indicators of genetic liability to ADHD and used in subsequent analyses. However, using PGS for MR purposes could be violating the MR relevance assumption especially when genetic variants associated at nominal values (e.g. p<0.5) rather than genome-wide significant variants are included. In addition, associations with PGS might also be capturing horizontal pleiotropic effects (i.e. shared genetic variants) and, unlike in a 2-sample MR framework, this is not possible to test formally.

For example, one study examined associations between PGS for ADHD and school data: ADHD PGS were associated with lower school grade attainment but strong evidence of association was not found for school absences (Hughes et al. 2021). This could reflect evidence because in this study PGS included only genetic variants strongly associated with ADHD (p<5x10^-8^), so the relevance MR assumption is satisfied. However, as with any PGS, it was not possible to test if the genetic variants have pleiotropic effects.

Taylor et al. (2018) examined associations between ADHD PGS using a range of p-value thresholds (<0.0005-0.05) and cohort study participation over time. ADHD PGS were associated with lower rates of participation for both mothers and children. While the study did not assess whether MR assumptions were met for these associations, the authors highlight important implications for MR studies, notably that incomplete participation in GWAS studies may lead to bias in MR estimates.

Most of the previously mentioned MR studies have been based on prior hypothesis testing. Another approach is to perform an MR phenome-wide association study (MR-pheWAS) which is a hypothesis-free analysis. In MR-pheWAS the genetic instrument for the exposure of interest is systematically tested with a potentially large number of phenotypes (Millard et al. 2015). Using a PGS for ADHD as an instrument, Leppert et al. (2020) performed a phenome scan in 334,976 UK Biobank participants. Several socio-demographic factors, general health and mental health, brain and cognition outcomes including younger age at first sexual intercourse and a history of physical maltreatment were associated with ADHD in UK Biobank indicating that ADHD could be having a potentially causal effect on them. However, despite using only genome-wide significant SNPs to construct the PGS, there could still be horizontal pleiotropic effects of the ADHD genetic variants and this is not possible to test for in this study design.

PGS for ADHD have widespread associations with other brain-based conditions, substance use, physical health and sociodemographic phenotypes. However, disentangling the causal effects of the genetic variants from pleiotropy is difficult when PGS are used.

***Summary data-based MR***

Summary data-based MR (SMR) extends the concept of MR by integrating summary-level GWAS data with other omic data, including expression quantitative trait loci (eQTL) and DNA methylation quantitative trait loci (mQTLs). When followed by the HEIDI (HEterogeneity In Dependent Instruments) test, the method can be potentially used to test if the effects of the genetic variants are mediated by expression or methylation levels. This is performed by distinguishing whether shared associated variants between, for example, eQTL data and GWAS data for ADHD, are due to linkage or pleiotropic effects (shared causal variants). The method cannot distinguish between pleiotropy or causal effects, so any effects that are not due to linkage could be attributed to either (Zhihong Zhu et al. 2016).

Gamazon et al. (2019) used multi-tissue transcriptome analyses followed by SMR/HEIDI for five neuropsychiatric diseases, including ADHD, to identify novel potentially causal genes in brain and non-brain tissues. Although SMR/HEIDI was not performed for ADHD, transcriptome analysis highlighted twelve independent genes associated with ADHD in different tissues including brain, whole blood, colon and adrenal gland. In another study, transcriptome-wide analysis followed by SMR/HEIDI revealed two genes associated with ADHD with potential evidence of shared causal variants. The expression levels of one of the genes were found to be different in a sample of Han Chinese children with ADHD and controls (Chen, Yao, Cai, Zhang, et al. 2022).

SMR followed by HEIDI was used to investigate overlap between fetal and adult eQTLs and mQTLs in brain and blood in ADHD (Hammerschlag et al. 2020). This study found multiple eQTLs and mQTLs in fetal brain, adult brain and blood associated with ADHD through pleiotropic effects at shared genetic variants. In another study, MR was used to test if gene expression in the brain could be mediating the effect of GWAS associated variants on eight brain-related phenotypes, including ADHD. Several genes whose expression could be causally linked to one of the phenotypes were identified. In addition, some of the genes showed evidence of multiple-trait colocalization, which means that there was evidence of shared causal variants between gene expression, methylation and the brain -related phenotypes.

SMR has started to provide insights on how the effects of genetic variants associated with ADHD could be mediated through expression or methylation in specific tissues. This method has the potential to identify biological mechanisms involved in ADHD.

***Other MR designs***

Other MR-based designs include a study by Nigg et al. (2016) which utilised a functional gene variant which regulates iron uptake and secondarily modulates lead metabolism. Consistent with a causal role of lead on ADHD, the gene variant moderated the association between blood level and ADHD.

Using another instrumental variable approach, within a dizygotic twin sample, Attermann et al. ( 2012) used co-twins sex of female twins as an instrumental variable for prenatal exposure to testosterone (i.e. male co-twin a proxy for higher prenatal exposure to testosterone to female to-twin). Inconsistent with a causal effect of prenatal exposure to testosterone on ADHD, female twins with a twin brother had lower parent-reported ADHD traits compared to those with a twin sister.

| **Supplementary Table 1a: Mendelian randomisation studies of ADHD and neurodevelopmental, mental health, personality and neurodegenerative conditions** | | | |
| --- | --- | --- | --- |
| Study | ADHD GWAS (Demontis et al. 2019) | Exposure/Outcome | Findings |
| Peyre et al. (2021) | N=53,293^*^ | Autism spectrum disorder (Grove et al. 2019): N=46,350 | Evidence of a causal effect of ADHD genetic liability on ASD and of a causal effect of ASD genetic liability on ADHD, both in the presence of horizontal pleiotropy and with heterogeneity. |
| Baranova et al. (2022) | N=55,374^**^ | Autism spectrum disorder, (Grove et al. 2019): N=46,350 | Evidence of a causal effect of ADHD genetic liability on ASD and of a causal effect of ASD genetic liability on ADHD, both with evidence of heterogeneity. |
| Riglin et al. (2021) | N=53,293^*^ | Depression (Wray et al. 2018; Howard et al. 2019): N=138,884-500,199 | Evidence of a causal effect of ADHD genetic liability on major depression but strong evidence not found for a broader depression phenotype (in the presence of horizontal pleiotropy). Strong evidence not found for a causal effect of major depression genetic liability on ADHD but evidence found for a broader depression phenotype (with evidence of heterogeneity). |
| Soler Artigas et al. (2022) | N=53,293^*^ | Depression, bipolar disorder and personality factors (N R Wray et al. 2018; Howard et al. 2019; Hou et al. 2016; Elsworth et al. 2017; Neale Lab, n.d.; Mats Nagel et al. 2018): N=23,895-449,019 | Investigated bidirectional effects for 124 traits. Evidence of causal effects of ADHD genetic liability on major depression; strong evidence not found for a broader definition of depression or for bipolar disorder. Strong evidence not found for effects in the other direction.  Examined associations with 17 personality factors, many relating to mental health. Evidence of a causal effect of ADHD genetic liability on four of these examined traits with evidence of a causal effect on ADHD for three of these plus an additional 9 traits. Only effects of ADHD genetic liability on frequency of unenthusiasm/disinterest in past 2 weeks and of genetic liability to able to confide and of frequency of tiredness/lethargy in last 2 weeks on ADHD met sensitivity analysis criteria. |
| Lim et al. (2020) | N=49,017^***^ | Self-harm (Neale Lab): N=125,925 | Evidence of a causal effect of ADHD genetic liability on self-harm: multivariable analyses suggested this was not independent of the causal effect of genetic liability to depression (N R Wray et al. 2018) on self-harm. |
| Zhang et al. (2021) | N=55,374^**^ | Neuroticism (M Nagel et al. 2018): (N=390,278) | Evidence of a causal effect of neuroticism genetic liability on ADHD; strong evidence not found for an effect of ADHD genetic liability on neuroticism. |
| Baranova et al. (2022) | N=55,374^**^ | Extraversion (van den Berg et al. 2016): N=63,030 | Evidence of a causal effect of extraversion genetic liability on ADHD, with evidence of heterogeneity. |
| ^*^European samples ^**^Full sample ^***^European samples, excluding sample overlap ^****^Excluding sample overlap | | | |

| **Supplementary Table 1b: Mendelian randomisation studies of ADHD and neurodevelopmental, mental health, personality and neurodegenerative conditions** | | | |
| --- | --- | --- | --- |
| Study | ADHD GWAS (Demontis et al. 2019) | Exposure/Outcome | Findings |
| Li et al. (2020) | N=55,374^**^ | Parkinson's Disease (Nalls et al. 2019): N=468,692 | Strong evidence not found of a causal effect of ADHD genetic liability on Parkinson’s Disease with little evidence of horizontal pleiotropy. Some evidence of a causal effect of Parkinson’s Disease genetic liability on ADHD. Similar results when adjusting for BMI (Yengo et al. 2018) and smoking (M. Liu et al. 2019). |
| Kim et al. (2021) | N=55,374^**^ | Parkinson's Disease (Simón-Sánchez et al. 2009): N=5,691 | Strong evidence not found of a causal effect of Parkinson’s Disease genetic liability on ADHD. |
| Pagoni et al. (2020) | N=55,374^**^ | Alzheimer’s disease (Jansen et al. 2019): N=455,258 | Strong evidence not found of a causal effect of ADHD genetic liability on Alzheimer’s disease or in the other direction, with limited evidence of horizontal pleiotropy or heterogeneity. Similar results when adjusting for educational attainment (Lee et al. 2018) and IQ (Savage et al. 2018). |
| ^*^European samples ^**^Full sample ^***^European samples, excluding sample overlap ^****^Excluding sample overlap | | | |

| **Supplementary Table 2a: Mendelian randomisation studies of ADHD and substance use** | | | |
| --- | --- | --- | --- |
| Study | ADHD GWAS (Demontis et al. 2019) | Exposure/Outcome | Findings |
| Fluharty, Sallis, and Munafò (2018) | N=55,374^**^ | Tobacco initiation (Tobacco and Consortium 2010): N=24,114-74,053 | Weak evidence of a causal effect of ADHD genetic liability on tobacco initiation (in the presence of horizontal pleiotropy); strong evidence not found of an effect on smoking age of onset. |
| Treur et al. (2021) | N=53,293^*^ | Multiple substance use measures (Liu et al. 2019; Wootton et al. 2020; Sanchez-Roige et al. 2018; Pasman et al. 2018; Cornelis et al. 2015): Ns=46,568-632,783 | Evidence of a causal effect of ADHD genetic liability on smoking initiation, cigarettes smoked per day, smoking cessation, lifetime smoking, cannabis use initiation and weak evidence for an effect on alcohol dependence; strong evidence not found for an effect on alcohol drinks per week, alcohol problems, or coffee consumption.  Weak evidence of a causal effect of genetic liability to smoking initiation on ADHD (in part due to horizontal pleiotropy); strong evidence not found for an effect of genetic liability to alcohol drinks per week, alcohol problems, alcohol dependence, cannabis use initiation or coffee consumption on ADHD. |
| Vilar-Ribó et al. (2021) | N=50,799^****^ | Multiple substance use measures (Liu et al. 2019; Walters et al. 2018; Pasman et al. 2018; Cabana-Domínguez et al. 2019; Neale Lab): Ns=1,917-631,790 | Evidence of a causal effect of ADHD genetic liability on smoking initiation, age of smoking initiation, cigarettes per day, lifetime cannabis use and (in the presence of horizontal pleiotropy) age of smoking initiation, but strong evidence not found for smoking cessation, alcohol dependence, cannabis dependence or ever addicted to illicit drugs.  Evidence of heterogeneity for smoking initiation and age of smoking initiation.  Evidence of causal effect on ADHD of genetic liability to smoking initiation and cannabis use, with heterogeneity and horizontal pleiotropy for smoking initiation. |
| Jang et al. (2022) | N=53,293^*^ | Multiple substance use measures (Liu et al. 2019): N=337,427-1,232,091 | Evidence of a causal effect of ADHD genetic liability on age of initiation of smoking and smoking initiation; strong evidence not found for an effect on cigarettes per day, smoking cessation, ever smoker, drinks per week or lifetime cannabis use.  Evidence of a causal effect of genetic liability to smoking initiation on ADHD; strong evidence not found for an effect of age of initiation of smoking, cigarettes per day, smoking cessation, ever smoker, drinks per week or lifetime cannabis use. |
| ^*^European samples ^**^Full sample ^***^European samples, excluding sample overlap ^****^Excluding sample overlap | | | |

| **Supplementary Table 2b: Mendelian randomisation studies of ADHD and substance use** | | | |
| --- | --- | --- | --- |
| Study | ADHD GWAS (Demontis et al. 2019) | Exposure/Outcome | Findings |
| Soler Artigas et al. (2022) | N=53,293^*^ | Multiple smoking and alcohol measures (Elsworth et al. 2017; Neale Lab, n.d.): N=23,332-462,434 | Investigated bidirectional effects for 124 traits including eight smoking and seven alcohol variables.  Evidence of causal effects of ADHD genetic liability on six smoking variables with bidirectional effects for three: effects of ADHD genetic liability on current tobacco use, ever smoked, pack years adult smoking as proportion of life span exposed to smoking and past tobacco smoking met sensitivity criteria as did effects of genetic liability to past tobacco smoking on ADHD.  Evidence of causal effects of ADHD genetic liability on six smoking variables with bidirectional effects for three: effects of genetic liability to ADHD on current tobacco use, ever smoked, pack years adult smoking as proportion of life span exposed to smoking and past tobacco smoking met sensitivity criteria as did effects of genetic liability to past tobacco smoking on ADHD. |
| Vink et al. (2021) | N=55,374^**^ | Nicotine dependence (Hancock et al. 2018): (N= 38,602) | Strong evidence not found of a causal effect of ADHD genetic liability on nicotine dependence. |
| Soler Artigas et al. (2020) | N=50,799^****^ | Cannabis (Stringer et al. 2016): N=32,330 | Evidence of a causal effect of ADHD genetic liability on cannabis with limited evidence of pleiotropy, heterogeneity or bidirectional associations. |
| ^*^European samples ^**^Full sample ^***^European samples, excluding sample overlap ^****^Excluding sample overlap | | | |

| **Supplementary Table 3a: Mendelian randomisation studies of ADHD and physical health and sleep** | | | |
| --- | --- | --- | --- |
| Study | ADHD GWAS (Demontis et al. 2019) | Exposure/Outcome | Findings |
| Leppert et al. (2021) | N=53,293^*^ | Physical health outcomes (Locke et al. 2015; Bradfield et al. 2012; Nikpay et al. 2015; Churchhouse and Neale; Scott et al. 2017; Abou-Khalil et al. 2018; Okada et al. 2014; Liu et al. 2015; Moffatt et al. 2010; Paternoster et al. 2015; Wang et al. 2014): Ns=13,848-337,159 | Evidence of a causal effect of ADHD genetic liability on childhood obesity, coronary artery disease, smoking and (in the presence of horizontal pleiotropy) inflammatory bowel disease; strong evidence not found for a causal effect on BMI, myocardial infarction, hypertension, systolic blood pressure, type 2 diabetes mellitus, migraine, epilepsy, autoimmune and allergic diseases and lung cancer.  Evidence of a bidirectional association with childhood obesity.  Multivariable MR suggested childhood obesity may mediate the impact of ADHD genetic liability on coronary artery disease. |
| Soler Artigas et al. (2022) | N=53,293^*^ | Multiple anthropometric measures, longevity, activity and sleep (Elsworth et al. 2017; Neale Lab, n.d.; Berndt et al. 2013; Wood et al. 2014; Shungin et al. 2015; Chan et al. 2015)(Pilling et al. 2017): N=16,067-462,400 | Investigated bidirectional effects for 124 traits including 20 anthropometric traits. Evidence of a causal effect of ADHD genetic liability on one anthropometric trait and evidence of genetic liability for 17 anthropometric traits having a causal effect on ADHD. After sensitivity analyses there was limited evidence of effects of ADHD genetic liability on the anthropomorphic traits and evidence of an effect of the genetic liability of eight traits (arm, leg, whole body and trunk fat-free mass, arm and trunk predicted mass, whole body water mass and weight) on ADHD.  Evidence of a causal effect of ADHD genetic liability on all 6 measures of longevity (decreasing maternal, paternal and combined age of death or attained age and decreasing the odds of both parents being in the top 10% of survival) although only effects on combined parental attained age met the sensitivity analysis criteria. Strong evidence was not found of a causal effect genetic liability to longevity on ADHD.  Evidence of a causal effect of ADHD genetic liability on four of eight activity measures with evidence of a causal effect on ADHD for two of them. After sensitivity analyses there was evidence of effects of ADHD genetic liability on frequency of stair climbing in last 4 weeks only. Strong evidence of a causal effects in either direction was not found for five sleep variables. |
| ^*^European samples ^**^Full sample ^***^European samples, excluding sample overlap ^****^Excluding sample overlap | | | |

| **Supplementary Table 3b: Mendelian randomisation studies of ADHD and physical health and sleep** | | | |
| --- | --- | --- | --- |
| Study | ADHD GWAS (Demontis et al. 2019) | Exposure/Outcome | Findings |
| Karhunen et al. (2021) | N=55,374^**^ | Obesity-related traits (Yengo et al. 2018; Neale Lab; Shungin et al. 2015): Ns= 224,459-688,566 | Evidence of a causal effect of ADHD genetic liability on higher BMI, waist circumference, waist-hip-ratio and BMI-adjusted waist-hip-ratio, but strong evidence not found for body fat percentage and basal metabolic rate. Evidence for horizontal pleiotropy for all outcomes except waist-hip-ratio.  Very little evidence with the negative control hair colour.  Evidence of causal effect of genetic liability to BMI on ADHD, waist circumference, body fat percentage and basal metabolic rate. |
| Hübel et al. (2019) | N=53,293^*^ | Body composition traits (Hübel et al. 2019) N=155,961 | Evidence of a causal effect of ADHD genetic liability on body mass index, fat mass or fat-free mass; strong evidence not found of a causal effect of ADHD genetic liability on body fat percentage. Similar pattern for males and females. |
| C.-Y. Liu et al. (2021) | N=55,374^**^ | BMI (Pulit et al. 2019): N=806,834 | Evidence of a causal effect of ADHD genetic liability on higher BMI with little evidence of horizontal pleiotropy.  Evidence of a causal effect of higher BMI genetic liability on ADHD (which remained but attenuated when controlling for education) with little evidence of horizontal pleiotropy. |
| Martins-Silva et (al. 2019) | N=55,374^**^ | BMI (Felix et al. 2016; Locke et al. 2015; Churchhouse and Neale): Ns=35,668-~337,000 | Evidence of a causal effect of ADHD genetic liability on higher childhood BMI and higher adult BMI for one sample with little evidence for another.  Evidence of a causal effect of higher childhood and adult BMI genetic liability on ADHD. |
| Zhaozhong Zhu et al. (2019) | N=53,293^*^ | Asthma (Zhaozhong Zhu et al. 2019): N=394,283 | Evidence of a causal effect of ADHD genetic liability on asthma; strong evidence not found of a causal effect of asthma genetic liability on ADHD. |
| Du et al. (2022) | N=53,293^*^ | Stroke and subtypes (Malik et al. 2018): N=446,969 | Evidence of a causal effect of ADHD genetic liability on higher risk of any ischemic stroke and large-artery atherosclerotic stroke. Multivariable analyses suggested that the causal effect of ADHD on stroke could be mediated through coronary artery disease. |
| Wang et al. (2020) | N=53,293^*^ | Mouth ulcers (Dudding et al. 2019): N=461,106 | Strong evidence not found of a causal effect of mouth ulcers genetic liability on ADHD or of ADHD genetic liability on mouth ulcers. |
| Peng et al. (2021) | N=53,293^*^ | Intracranial Aneurysms (Bakker et al. 2020): N=79,429 | Strong evidence not found of a causal effect of intracranial aneurysms genetic liability on ADHD or of ADHD genetic liability on intracranial aneurysms. |
| ^*^European samples ^**^Full sample ^***^European samples, excluding sample overlap ^****^Excluding sample overlap | | | |

| **Supplementary Table 3c: Mendelian randomisation studies of ADHD and physical health and sleep** | | | |
| --- | --- | --- | --- |
| Study | ADHD GWAS (Demontis et al. 2019) | Exposure/Outcome | Findings |
| Liu et al. (2021) | N=55,374^**^ | Covid-19(COVID-19 Host Genetics Initiative): N=1,388,342-1,887,658 | Evidence of a causal effect of ADHD genetic liability on hospitalised Covid-19; strong evidence not found for an effect on critically ill COVID-19.  Strong evidence not found for an effect of genetic liability to hospitalised Covid-19 or critically ill COVID-19 on ADHD. |
| Gao et al. (2019) | N=55,374^**^ | Insomnia (Jansen et al. 2019): N=386,533 | Strong evidence not found of a causal effect of insomnia genetic liability on ADHD or of ADHD genetic liability on insomnia. |
| Sun et al. (2022) | N=53,293^*^ | Insomnia, chronotype, sleep duration (Dashti et al. 2019; P. R. Jansen et al. 2019; Jones et al. 2019): N=446,118- 1,331,010 | Evidence of causal effect of genetic liability to insomnia on ADHD. Strong evidence not found of a causal effect of chronotype or sleep duration genetic liability on ADHD. Suggestive evidence of ADHD genetic liability on sleep duration. |
| Carpena et al. (2021) | N=55,374^**^ | Sleep-related traits (Jones et al. 2019; Dashti et al. 2019; Lane et al. 2019; Wang et al. 2019; Jansen et al. 2019): Ns=10,554- 1,331,010 | Evidence of a causal effect of ADHD genetic liability on longer sleep duration and chronotype (in the presence of pleiotropy), but strong evidence not found for insomnia, snoring, daytime napping, daytime sleepiness or ease getting up.  Evidence of causal effects of sleep-related traits genetic liability on ADHD for insomnia, daytime napping and shorter sleep duration, but strong evidence not found for chronotype, daytime sleepiness, daytime dozing, ease getting up or snoring. |
| ^*^European samples ^**^Full sample ^***^European samples, excluding sample overlap ^****^Excluding sample overlap | | | |

| **Supplementary Table 4: Mendelian randomisation studies of ADHD and intelligence and indicators of socio-economic status** | | | |
| --- | --- | --- | --- |
| Study | ADHD GWAS (Demontis et al. 2019) | Exposure/Outcome | Findings |
| Savage et al. (2018) | N=53,293^*^ | Intelligence (Savage et al. 2018): N=269,867 | Evidence of a causal effect of lower intelligence genetic liability on ADHD and of ADHD genetic liability on lower intelligence. |
| Michaëlsson et al. (2022) | N=55,374^**^ | Socioeconomic status (IEU Open GWAS Project; Plana-Ripoll et al. 2019; Savage et al. 2018): N=269,867-766,345 | Evidence of a causal effect of genetic liability to lower education, lower household income (evidence of horizontal pleiotropy) and higher Townsend deprivation index on ADHD, independent of intelligence. Weaker evidence of a causal effect of genetic liability to intelligence on ADHD, which was not independent of education.  Evidence of a causal effect of ADHD genetic liability on lower education, lower household income and higher Townsend deprivation index on ADHD; strong evidence not found for an effect on intelligence. |
| Rao et al. (2022) | N=55,374^**^ | Intelligence (Savage et al. 2018): N=269,867 | Evidence of a causal effect of lower intelligence genetic liability on ADHD. |
| Soler Artigas et al. (2022) | N=53,293^*^ | Intelligence, educational attainment, employment and household variables (Elsworth et al. 2017; Neale Lab, n.d.; Lee et al. 2018; Savage et al. 2018): N=149,051-766,345 | Investigated bidirectional effects for 124 traits. Evidence of a causal effect of ADHD genetic liability on three of five examined measures of cognitive function and intelligence with evidence of a causal effect on ADHD for two of these plus the additional two examined traits. Only the effect of ADHD genetic liability to cognitive performance survived sensitivity analyses but showed high heterogeneity.  Evidence of causal effects of ADHD genetic liability on all four examined measures of education and employment, and of genetic liability to these on ADHD. Effects in both direction survived sensitivity analyses for age completed full-time education and years of schooling.  Evidence of causal effects of ADHD genetic liability on five of nine examined household measures: causal evidence on income, deprivation and number of sisters passed sensitivity analyses. Evidence of causal effects of genetic liability to three of nine examined household measures on ADHD: effect of deprivation passed sensitivity analyses. |
| Dardani et al. (2021) | N=55,374^**^ | Educational attainment (Lee et al. 2018): N=1,131,881 | Evidence of a causal effect of ADHD genetic liability on lower educational attainment, independently of cognitive ability (Savage et al. 2018). Evidence of a causal effect of higher educational attainment genetic liability on lower risk of ADHD, independently of cognitive ability. |
| ^*^European samples ^**^Full sample ^***^European samples, excluding sample overlap ^****^Excluding sample overlap | | | |

| **Supplementary Table 5: Mendelian randomisation studies of ADHD and birthweight, gestational age and environmental exposures** | | | |
| --- | --- | --- | --- |
| Study | ADHD GWAS (Demontis et al. 2019) | Exposure/Outcome | Findings |
| Arafat and Minică (2018) | N=55,374^**^ | Birth weight (Horikoshi et al. 2016): N=153,781 | Strong evidence not found of a causal effect of genetic liability to low birth weight on ADHD. |
| Orri et al. (2021) | N=53,293^*^ | Birth weight (Warrington et al. 2019): N=264,498 | Evidence of a causal effect of genetic liability to low birth weight on ADHD. |
| Soler Artigas et al. (2022) | N=53,293^*^ | Birth weight, breastfed, maternal smoking around birth, place of birth and multiple environmental measures (Elsworth et al. 2017; Neale Lab): N=261,932-462,433 | Investigated bidirectional effects for 124 traits. Evidence of a causal effect of ADHD genetic liability on maternal smoking around birth and of genetic liability to maternal smoking around birth on ADHD although only the former met sensitivity criteria. Strong evidence was not found in either direction for birth weight, being breastfed as a baby or place of birth in UK.  Evidence of causal effects of ADHD genetic liability on nine of 18 additional environmental measures: two measures of air pollution and two measures of sexual behaviour passed sensitivity analyses. Evidence of causal effects of genetic liability to eight of the 18 measures on ADHD: effect of age of first sexual intercourse, length of mobile phone use and time spent watching television passed sensitivity analyses. |
| Yao et al. (2022) | N=53,293^*^ | Gestational age (X. Liu et al. 2019): N=84,689 | Strong evidence not found of a causal effect of genetic liability to gestational duration on ADHD. |
| Warrier et al. (2021) | N=53,293^*^ | Childhood maltreatment (Warrier et al. 2021): N=185,414 | Evidence of a causal effect of ADHD genetic liability on childhood maltreatment and of childhood maltreatment genetic liability on ADHD. |
| Wendt et al. (2019) | N=53,293^*^ | Computerized device use (Wendt et al. 2019): N=301,157 | Evidence of a causal effect of ADHD genetic liability on phone use and of genetic liability to phone use on ADHD. |
| ^*^European samples ^**^Full sample ^***^European samples, excluding sample overlap ^****^Excluding sample overlap | | | |

| **Supplementary Table 6: Mendelian randomisation studies of ADHD and biological markers** | | | |
| --- | --- | --- | --- |
| Study | ADHD GWAS (Demontis et al. 2019) | Exposure/Outcome | Findings |
| Yang et al. (2020) | N=55,374^**^ | Human Serum Metabolites (Yang et al. 2020): (N=7,824) | Examined associations between 486 metabolites on ADHD; evidence of a causal effect for 27: one passed Bonferroni correction (1-docosahexaenoylglycerophosphocholine). |
| Yang et al. (2022) | N=55,374^**^ | Plasma proteins (B. B. Sun et al. 2018): N=3,301 | Examined associations between 2,994 plasma protein on ADHD; evidence of a causal effect for one (MANBA). |
| Chen, Yao, Cai, Fu, et al. (2022) | N=55,374^**^ | Systemic inflammatory regulators (Ahola-Olli et al. 2017): N=8,293 | Examined associations between 41 systemic inflammatory regulators and ADHD. Weak evidence of a causal effect of genetic liability to two systemic inflammatory regulators (beta nerve growth factor and stem cell factor) on ADHD and weak evidence of a causal effect of ADHD genetic liability on two systemic inflammatory regulators (interleukin7 and tumour necrosis factor–alpha). |
| Zhao et al. (2021) | N=53,293^*^ | Serum urate levels (Köttgen et al. 2013): N=110,347 | Strong evidence not found of a causal effect of Serum urate levels genetic liability on ADHD. |
| Libuda et al. (2021) | N=53,293^*^ | 25(OH)vitamin D levels (Jiang et al. 2018): N= 79,366 | Strong evidence not found of a causal effect of 25(OH)D genetic liability on ADHD or of ADHD genetic liability on 25(OH)D. |
| ^*^European samples ^**^Full sample ^***^European samples, excluding sample overlap ^****^Excluding sample overlap | | | |

**References**

Abou-Khalil, Bassel, Pauls Auce, Andreja Avbersek, Melanie Bahlo, David J Balding, Thomas Bast, Larry Baum, et al. 2018. “Genome-Wide Mega-Analysis Identifies 16 Loci and Highlights Diverse Biological Mechanisms in the Common Epilepsies.” *Nature Communications* 9 (1): 5269. https://doi.org/10.1038/s41467-018-07524-z.

Ahola-Olli, Ari V, Peter Würtz, Aki S Havulinna, Kristiina Aalto, Niina Pitkänen, Terho Lehtimäki, Mika Kähönen, Leo-Pekka Lyytikäinen, Emma Raitoharju, and Ilkka Seppälä. 2017. “Genome-Wide Association Study Identifies 27 Loci Influencing Concentrations of Circulating Cytokines and Growth Factors.” *The American Journal of Human Genetics* 100 (1): 40–50.

Arafat, Subhi, and Camelia C Minică. 2018. “Fetal Origins of Mental Disorders? An Answer Based on Mendelian Randomization.” *Twin Research and Human Genetics* 21 (6): 485–94. https://doi.org/DOI: 10.1017/thg.2018.65.

Attermann, Jørn, Carsten Obel, Niels Bilenberg, Claudia Maria Nordenbæk, Axel Skytthe, and Jørn Olsen. 2012. “Traits of ADHD and Autism in Girls with a Twin Brother: A Mendelian Randomization Study.” *European Child & Adolescent Psychiatry* 21 (9): 503–9. https://doi.org/10.1007/s00787-012-0287-4.

Bakker, Mark K, Rick A A van der Spek, Wouter van Rheenen, Sandrine Morel, Romain Bourcier, Isabel C Hostettler, Varinder S Alg, et al. 2020. “Genome-Wide Association Study of Intracranial Aneurysms Identifies 17 Risk Loci and Genetic Overlap with Clinical Risk Factors.” *Nature Genetics* 52 (12): 1303–13. https://doi.org/10.1038/s41588-020-00725-7.

Baranova, Ancha, Jun Wang, Hongbao Cao, Jiang-Huan Chen, Jiu Chen, Miao Chen, Sulin Ni, et al. 2022. “Shared Genetics between Autism Spectrum Disorder and Attention-Deficit/Hyperactivity Disorder and Their Association with Extraversion.” *Psychiatry Research* 314: 114679. https://doi.org/https://doi.org/10.1016/j.psychres.2022.114679.

Berg, Stéphanie M van den, Marleen H M de Moor, Karin J H Verweij, Robert F Krueger, Michelle Luciano, Alejandro Arias Vasquez, Lindsay K Matteson, et al. 2016. “Meta-Analysis of Genome-Wide Association Studies for Extraversion: Findings from the Genetics of Personality Consortium.” *Behavior Genetics* 46 (2): 170–82. https://doi.org/10.1007/s10519-015-9735-5.

Berndt, Sonja I, Stefan Gustafsson, Reedik Mägi, Andrea Ganna, Eleanor Wheeler, Mary F Feitosa, Anne E Justice, Keri L Monda, Damien C Croteau-Chonka, and Felix R Day. 2013. “Genome-Wide Meta-Analysis Identifies 11 New Loci for Anthropometric Traits and Provides Insights into Genetic Architecture.” *Nature Genetics* 45 (5): 501–12.

Bradfield, Jonathan P, H Rob Taal, Nicholas J Timpson, André Scherag, Cecile Lecoeur, Nicole M Warrington, Elina Hypponen, Claus Holst, Beatriz Valcarcel, and Elisabeth Thiering. 2012. “A Genome-Wide Association Meta-Analysis Identifies New Childhood Obesity Loci.” *Nature Genetics* 44 (5): 526.

Cabana-Domínguez, Judit, Anu Shivalikanjli, Noèlia Fernàndez-Castillo, and Bru Cormand. 2019. “Genome-Wide Association Meta-Analysis of Cocaine Dependence: Shared Genetics with Comorbid Conditions.” *Progress in Neuro-Psychopharmacology and Biological Psychiatry* 94: 109667.

Carpena, Marina Xavier, Carolina Bonilla, Alicia Matijasevich, Thais Martins-Silva, Julia P Genro, Mara Helena Hutz, Luis Augusto Rohde, and Luciana Tovo-Rodrigues. 2021. “Sleep-Related Traits and Attention-Deficit/Hyperactivity Disorder Comorbidity: Shared Genetic Risk Factors, Molecular Mechanisms, and Causal Effects.” *The World Journal of Biological Psychiatry* 22 (10): 778–91. https://doi.org/10.1080/15622975.2021.1907719.

Chan, Yingleong, Rany M Salem, Yu-Han H Hsu, George McMahon, Tune H Pers, Sailaja Vedantam, Tonu Esko, Michael H Guo, Elaine T Lim, and Lude Franke. 2015. “Genome-Wide Analysis of Body Proportion Classifies Height-Associated Variants by Mechanism of Action and Implicates Genes Important for Skeletal Development.” *The American Journal of Human Genetics* 96 (5): 695–708.

Chen, Xinzhen, Ting Yao, Jinliang Cai, Xihang Fu, Huiru Li, and Jing Wu. 2022. “Systemic Inflammatory Regulators and 7 Major Psychiatric Disorders: A Two-Sample Mendelian Randomization Study.” *Progress in Neuro-Psychopharmacology and Biological Psychiatry* 116: 110534. https://doi.org/https://doi.org/10.1016/j.pnpbp.2022.110534.

Chen, Xinzhen, Ting Yao, Jinliang Cai, Qi Zhang, Shanyawen Li, Huiru Li, Xihang Fu, and Jing Wu. 2022. “A Novel Cis-Regulatory Variant Modulating TIE1 Expression Associated with Attention Deficit Hyperactivity Disorder in Han Chinese Children.” *Journal of Affective Disorders* 300: 179–88. https://doi.org/https://doi.org/10.1016/j.jad.2021.12.066.

Churchhouse, C, and B Neale. n.d. “Rapid GWAS of Thousands of Phenotypes for 337, 000 Samples in the UK Biobank.” Accessed May 4, 2020. http://www.nealelab.is/blog/2017/7/19/rapid-gwas-of-thousands-of-phenotypes-for-337000-samples-in-the-uk-biobank.

Cornelis, M C, E M Byrne, T Esko, M A Nalls, A Ganna, N Paynter, K L Monda, et al. 2015. “Genome-Wide Meta-Analysis Identifies Six Novel Loci Associated with Habitual Coffee Consumption.” *Molecular Psychiatry* 20 (5): 647–56. https://doi.org/10.1038/mp.2014.107.

COVID-19 Host Genetics Initiative. n.d. “Genome-Wide Association Study.” https://www.covid19hg.org/results/r5/.

Dardani, Christina, Lucy Riglin, Beate Leppert, Eleanor Sanderson, Dheeraj Rai, Laura D Howe, George Davey Smith, et al. 2021. “Is Genetic Liability to ADHD and ASD Causally Linked to Educational Attainment?” *International Journal of Epidemiology* 50 (6): 2011–23. https://doi.org/10.1093/ije/dyab107.

Dashti, Hassan S, Samuel E Jones, Andrew R Wood, Jacqueline M Lane, Vincent T Van Hees, Heming Wang, Jessica A Rhodes, Yanwei Song, Krunal Patel, and Simon G Anderson. 2019. “Genome-Wide Association Study Identifies Genetic Loci for Self-Reported Habitual Sleep Duration Supported by Accelerometer-Derived Estimates.” *Nature Communications* 10 (1): 1–12.

Demontis, Ditte, Raymond K. Walters, Joanna Martin, Manuel Mattheisen, Thomas D. Als, Esben Agerbo, Gísli Baldursson, et al. 2019. “Discovery of the First Genome-Wide Significant Risk Loci for Attention Deficit/Hyperactivity Disorder.” *Nature Genetics* 51 (1): 63–75. https://doi.org/10.1038/s41588-018-0269-7.

Du, Runming, Yi Zhou, Chong You, Kevin Liu, Daniel A King, Zhi-Sheng Liang, Janice M Ranson, David J Llewellyn, Jie Huang, and Zhenyu Zhang. 2022. “Attention-Deficit/Hyperactivity Disorder and Ischemic Stroke: A Mendelian Randomization Study.” *International Journal of Stroke*, June, 17474930221108272. https://doi.org/10.1177/17474930221108272.

Dudding, Tom, Simon Haworth, Penelope A Lind, J Fah Sathirapongsasuti, Joyce Y Tung, Ruth Mitchell, Lucía Colodro-Conde, Sarah E Medland, Scott Gordon, and Benjamin Elsworth. 2019. “Genome Wide Analysis for Mouth Ulcers Identifies Associations at Immune Regulatory Loci.” *Nature Communications* 10 (1): 1–12.

Elsworth, Ben, Ruth Mitchell, C A Raistrick, Lavinia Paternoster, Gibran Hemani, and Tom Gaunt. 2017. “MRC IEU UK Biobank GWAS Pipeline Version 1.” *Bristol, UK: University of Bristol*.

Felix, Janine F, Jonathan P Bradfield, Claire Monnereau, Ralf J P Van Der Valk, Evie Stergiakouli, Alessandra Chesi, Romy Gaillard, Bjarke Feenstra, Elisabeth Thiering, and Eskil Kreiner-Møller. 2016. “Genome-Wide Association Analysis Identifies Three New Susceptibility Loci for Childhood Body Mass Index.” *Human Molecular Genetics* 25 (2): 389–403.

Fluharty, Meg E, Hannah Sallis, and Marcus R Munafò. 2018. “Investigating Possible Causal Effects of Externalizing Behaviors on Tobacco Initiation: A Mendelian Randomization Analysis.” *Drug and Alcohol Dependence* 191: 338–42. https://doi.org/https://doi.org/10.1016/j.drugalcdep.2018.07.015.

Gamazon, Eric R, Aeilko H Zwinderman, Nancy J Cox, Damiaan Denys, and Eske M Derks. 2019. “Multi-Tissue Transcriptome Analyses Identify Genetic Mechanisms Underlying Neuropsychiatric Traits.” *Nature Genetics* 51 (6): 933–40. https://doi.org/10.1038/s41588-019-0409-8.

Gao, Xue, Ling-Xian Meng, Kai-Li Ma, Jie Liang, Hui Wang, Qian Gao, and Tong Wang. 2019. “The Bidirectional Causal Relationships of Insomnia with Five Major Psychiatric Disorders: A Mendelian Randomization Study.” *European Psychiatry* 60: 79–85. https://doi.org/DOI: 10.1016/j.eurpsy.2019.05.004.

Grove, Jakob, Stephan Ripke, Thomas D Als, Manuel Mattheisen, Raymond K Walters, Hyejung Won, Jonatan Pallesen, et al. 2019. “Identification of Common Genetic Risk Variants for Autism Spectrum Disorder.” *Nature Genetics* 51 (3): 431–44. https://doi.org/10.1038/s41588-019-0344-8.

Hammerschlag, Anke R, Enda M Byrne, Mawussé Agbessi, Habibul Ahsan, Isabel Alves, Anand Andiappan, Wibowo Arindrarto, et al. 2020. “Refining Attention-Deficit/Hyperactivity Disorder and Autism Spectrum Disorder Genetic Loci by Integrating Summary Data From Genome-Wide Association, Gene Expression, and DNA Methylation Studies.” *Biological Psychiatry* 88 (6): 470–79. https://doi.org/https://doi.org/10.1016/j.biopsych.2020.05.002.

Hancock, D. B., Y. Guo, G. W. Reginsson, N. C. Gaddis, S. M. Lutz, R. Sherva, A. Loukola, et al. 2018. “Genome-Wide Association Study across European and African American Ancestries Identifies a SNP in DNMT3B Contributing to Nicotine Dependence.” *Molecular Psychiatry* 23 (9): 1911–19. https://doi.org/10.1038/mp.2017.193.

Horikoshi, M, R N Beaumont, F R Day, N M Warrington, M N Kooijman, J Fernandez-Tajes, B Feenstra, et al. 2016. “Genome-Wide Associations for Birth Weight and Correlations with Adult Disease.” *Nature* 538 (7624): 248–52. https://doi.org/10.1038/nature19806.

Hou, Liping, Sarah E Bergen, Nirmala Akula, Jie Song, Christina M Hultman, Mikael Landén, Mazda Adli, et al. 2016. “Genome-Wide Association Study of 40,000 Individuals Identifies Two Novel Loci Associated with Bipolar Disorder.” *Human Molecular Genetics* 25 (15): 3383–94. https://doi.org/10.1093/hmg/ddw181.

Howard, David M., Mark J. Adams, Toni Kim Clarke, Jonathan D. Hafferty, Jude Gibson, Masoud Shirali, Jonathan R.I. Coleman, et al. 2019. “Genome-Wide Meta-Analysis of Depression Identifies 102 Independent Variants and Highlights the Importance of the Prefrontal Brain Regions.” *Nature Neuroscience* 22 (3): 343–52. https://doi.org/10.1038/s41593-018-0326-7.

Hübel, Christopher, Héléna A Gaspar, Jonathan R I Coleman, Ken B Hanscombe, Kirstin Purves, Inga Prokopenko, Mariaelisa Graff, et al. 2019. “Genetic Correlations of Psychiatric Traits with Body Composition and Glycemic Traits Are Sex- and Age-Dependent.” *Nature Communications* 10 (1): 5765. https://doi.org/10.1038/s41467-019-13544-0.

Hughes, Amanda, Kaitlin H Wade, Matt Dickson, Frances Rice, Alisha Davies, Neil M Davies, and Laura D Howe. 2021. “Common Health Conditions in Childhood and Adolescence, School Absence, and Educational Attainment: Mendelian Randomization Study.” *Npj Science of Learning* 6 (1): 1. https://doi.org/10.1038/s41539-020-00080-6.

IEU Open GWAS Project. n.d. “UK Biobank GWAS.”

Jang, Seon-Kyeong, Gretchen Saunders, MengZhen Liu, Yu Jiang, Dajiang J Liu, and Scott Vrieze. 2022. “Genetic Correlation, Pleiotropy, and Causal Associations between Substance Use and Psychiatric Disorder.” *Psychological Medicine* 52 (5): 968–78. https://doi.org/DOI: 10.1017/S003329172000272X.

Jansen, Iris E, Jeanne E Savage, Kyoko Watanabe, Julien Bryois, Dylan M Williams, Stacy Steinberg, Julia Sealock, et al. 2019. “Genome-Wide Meta-Analysis Identifies New Loci and Functional Pathways Influencing Alzheimer’s Disease Risk.” *Nature Genetics* 51 (3): 404–13. https://doi.org/10.1038/s41588-018-0311-9.

Jansen, Philip R, Kyoko Watanabe, Sven Stringer, Nathan Skene, Julien Bryois, Anke R Hammerschlag, Christiaan A de Leeuw, Jeroen S Benjamins, Ana B Muñoz-Manchado, and Mats Nagel. 2019. “Genome-Wide Analysis of Insomnia in 1,331,010 Individuals Identifies New Risk Loci and Functional Pathways.” *Nature Genetics* 51 (3): 394–403.

Jiang, Xia, Paul F O’Reilly, Hugues Aschard, Yi-Hsiang Hsu, J Brent Richards, Josée Dupuis, Erik Ingelsson, et al. 2018. “Genome-Wide Association Study in 79,366 European-Ancestry Individuals Informs the Genetic Architecture of 25-Hydroxyvitamin D Levels.” *Nature Communications* 9 (1): 260. https://doi.org/10.1038/s41467-017-02662-2.

Jones, S E, J M Lane, A R Wood, V T Van Hees, J Tyrrell, R N Beaumont, A R Jeffries, H S Dashti, M Hillsdon, and K S Ruth. 2019. “Genome-Wide Association Analyses of Chronotype in 697,828 Individuals Provides Insights into Circadian Rhythms. Nat Commun 2019; 10: 343.” S41467-018-08259-7.

Karhunen, Ville, Tom A Bond, Verena Zuber, Tuula Hurtig, Irma Moilanen, Marjo-Riitta Järvelin, Marina Evangelou, and Alina Rodriguez. 2021. “The Link between Attention Deficit Hyperactivity Disorder (ADHD) Symptoms and Obesity-Related Traits: Genetic and Prenatal Explanations.” *Translational Psychiatry* 11 (1): 455. https://doi.org/10.1038/s41398-021-01584-4.

Kim, Kiwon, Soyeon Kim, Woojae Myung, Injeong Shim, Hyewon Lee, Beomsu Kim, Sung K Cho, Joohyun Yoon, Doh K Kim, and Hong-Hee Won. 2021. “Shared Genetic Background between Parkinson’s Disease and Schizophrenia: A Two-Sample Mendelian Randomization Study.” *Brain Sciences* . https://doi.org/10.3390/brainsci11081042.

Köttgen, Anna, Eva Albrecht, Alexander Teumer, Veronique Vitart, Jan Krumsiek, Claudia Hundertmark, Giorgio Pistis, Daniela Ruggiero, Conall M O’Seaghdha, and Toomas Haller. 2013. “Genome-Wide Association Analyses Identify 18 New Loci Associated with Serum Urate Concentrations.” *Nature Genetics* 45 (2): 145–54.

Lane, Jacqueline M, Samuel E Jones, Hassan S Dashti, Andrew R Wood, Krishna G Aragam, Vincent T van Hees, Linn B Strand, Bendik S Winsvold, Heming Wang, and Jack Bowden. 2019. “Biological and Clinical Insights from Genetics of Insomnia Symptoms.” *Nature Genetics* 51 (3): 387–93.

Lee, James J, Robbee Wedow, Aysu Okbay, Edward Kong, Omeed Maghzian, Meghan Zacher, Tuan Anh Nguyen-Viet, Peter Bowers, Julia Sidorenko, and Richard Karlsson Linnér. 2018. “Gene Discovery and Polygenic Prediction from a 1.1-Million-Person GWAS of Educational Attainment.” *Nature Genetics* 50 (8): 1112.

Leppert, Beate, Louise A.C. Millard, Lucy Riglin, George Davey Smith, Anita Thapar, Kate Tilling, Esther Walton, and Evie Stergiakouli. 2020. “A Cross-Disorder PRS-PheWAS of 5 Major Psychiatric Disorders in UK Biobank.” *PLoS Genetics* 16 (5). https://doi.org/10.1371/JOURNAL.PGEN.1008185.

Leppert, Beate, Lucy Riglin, Robyn E Wootton, Christina Dardani, Ajay Thapar, James R Staley, Kate Tilling, George Davey Smith, Anita Thapar, and Evie Stergiakouli. 2021. “The Effect of Attention Deficit/Hyperactivity Disorder on Physical Health Outcomes: A 2-Sample Mendelian Randomization Study.” *American Journal of Epidemiology* 190 (6): 1047–55. https://doi.org/10.1093/aje/kwaa273.

Li, Gloria Hoi-Yee, Grace Mengqin Ge, Ching-Lung Cheung, Patrick Ip, David Coghill, and Ian Chi-Kei Wong. 2020. “Evaluation of Causality between ADHD and Parkinson’s Disease: Mendelian Randomization Study.” *European Neuropsychopharmacology* 37: 49–63. https://doi.org/https://doi.org/10.1016/j.euroneuro.2020.06.001.

Libuda, Lars, Roaa Naaresh, Christine Ludwig, Björn-Hergen Laabs, Jochen Antel, Manuel Föcker, Johannes Hebebrand, Anke Hinney, and Triinu Peters. 2021. “A Mendelian Randomization Study on Causal Effects of 25(OH)Vitamin D Levels on Attention Deficit/Hyperactivity Disorder.” *European Journal of Nutrition* 60 (5): 2581–91. https://doi.org/10.1007/s00394-020-02439-2.

Lim, Kai Xiang, Frühling Rijsdijk, Saskia P Hagenaars, Adam Socrates, Shing Wan Choi, Jonathan R I Coleman, Kylie P Glanville, Cathryn M Lewis, and Jean-Baptiste Pingault. 2020. “Studying Individual Risk Factors for Self-Harm in the UK Biobank: A Polygenic Scoring and Mendelian Randomisation Study.” *PLoS Medicine* 17 (6): e1003137.

Liu, Chao-Yu, Tabea Schoeler, Neil M Davies, Hugo Peyre, Kai-Xiang Lim, Edward D Barker, Clare Llewellyn, Frank Dudbridge, and Jean-Baptiste Pingault. 2021. “Are There Causal Relationships between Attention-Deficit/Hyperactivity Disorder and Body Mass Index? Evidence from Multiple Genetically Informed Designs.” *International Journal of Epidemiology* 50 (2): 496–509. https://doi.org/10.1093/ije/dyaa214.

Liu, Jimmy Z, Suzanne Van Sommeren, Hailiang Huang, Siew C Ng, Rudi Alberts, Atsushi Takahashi, Stephan Ripke, James C Lee, Luke Jostins, and Tejas Shah. 2015. “Association Analyses Identify 38 Susceptibility Loci for Inflammatory Bowel Disease and Highlight Shared Genetic Risk across Populations.” *Nature Genetics* 47 (9): 979–86.

Liu, Mengzhen, Yu Jiang, Robbee Wedow, Yue Li, David M Brazel, Fang Chen, Gargi Datta, Jose Davila-Velderrain, Daniel McGuire, and Chao Tian. 2019. “Association Studies of up to 1.2 Million Individuals Yield New Insights into the Genetic Etiology of Tobacco and Alcohol Use.” *Nature Genetics* 51 (2): 237–44.

Liu, Ningning, Jiang-Shan Tan, Lu Liu, Yufeng Wang, Lu Hua, and Qiujin Qian. 2021. “Genetic Predisposition Between COVID-19 and Four Mental Illnesses: A Bidirectional, Two-Sample Mendelian Randomization Study.” *Frontiers in Psychiatry*. https://www.frontiersin.org/article/10.3389/fpsyt.2021.746276.

Liu, Xueping, Dorte Helenius, Line Skotte, Robin N Beaumont, Matthias Wielscher, Frank Geller, Julius Juodakis, Anubha Mahajan, Jonathan P Bradfield, and Frederick T J Lin. 2019. “Variants in the Fetal Genome near Pro-Inflammatory Cytokine Genes on 2q13 Associate with Gestational Duration.” *Nature Communications* 10 (1): 1–13.

Locke, Adam E, Bratati Kahali, Sonja I Berndt, Anne E Justice, Tune H Pers, Felix R Day, Corey Powell, Sailaja Vedantam, Martin L Buchkovich, and Jian Yang. 2015. “Genetic Studies of Body Mass Index Yield New Insights for Obesity Biology.” *Nature* 518 (7538): 197–206.

Malik, Rainer, Ganesh Chauhan, Matthew Traylor, Muralidharan Sargurupremraj, Yukinori Okada, Aniket Mishra, Loes Rutten-Jacobs, et al. 2018. “Multiancestry Genome-Wide Association Study of 520,000 Subjects Identifies 32 Loci Associated with Stroke and Stroke Subtypes.” *Nature Genetics* 50 (4): 524–37. https://doi.org/10.1038/s41588-018-0058-3.

Martins-Silva, Thais, Juliana dos Santos Vaz, Mara Helena Hutz, Angélica Salatino-Oliveira, Júlia Pasqualini Genro, Fernando Pires Hartwig, Carlos Renato Moreira-Maia, Luis Augusto Rohde, Maria Carolina Borges, and Luciana Tovo-Rodrigues. 2019. “Assessing Causality in the Association between Attention-Deficit/Hyperactivity Disorder and Obesity: A Mendelian Randomization Study.” *International Journal of Obesity* 43 (12): 2500–2508. https://doi.org/10.1038/s41366-019-0346-8.

Michaëlsson, Madeleine, Shuai Yuan, Håkan Melhus, John A Baron, Liisa Byberg, Susanna C Larsson, and Karl Michaëlsson. 2022. “The Impact and Causal Directions for the Associations between Diagnosis of ADHD, Socioeconomic Status, and Intelligence by Use of a Bi-Directional Two-Sample Mendelian Randomization Design.” *BMC Medicine* 20 (1): 106. https://doi.org/10.1186/s12916-022-02314-3.

Millard, Louise A C, Neil M Davies, Nic J Timpson, Kate Tilling, Peter A Flach, and George Davey Smith. 2015. “MR-PheWAS: Hypothesis Prioritization among Potential Causal Effects of Body Mass Index on Many Outcomes, Using Mendelian Randomization.” *Scientific Reports* 5 (1): 16645. https://doi.org/10.1038/srep16645.

Moffatt, Miriam F, Ivo G Gut, Florence Demenais, David P Strachan, Emmanuelle Bouzigon, Simon Heath, Erika von Mutius, Martin Farrall, Mark Lathrop, and William O C M Cookson. 2010. “A Large-Scale, Consortium-Based Genomewide Association Study of Asthma.” *New England Journal of Medicine* 363 (13): 1211–21.

Nagel, M, P R Jansen, S Stringer, K Watanabe, C A de Leeuw, J Bryois, J E Savage, et al. 2018. “Meta-Analysis of Genome-Wide Association Studies for Neuroticism in 449,484 Individuals Identifies Novel Genetic Loci and Pathways.” *Nat Genet* 50 (7): 920–27. https://doi.org/10.1038/s41588-018-0151-7.

Nagel, Mats, Kyoko Watanabe, Sven Stringer, Danielle Posthuma, and Sophie van der Sluis. 2018. “Item-Level Analyses Reveal Genetic Heterogeneity in Neuroticism.” *Nature Communications* 9 (1): 905. https://doi.org/10.1038/s41467-018-03242-8.

Nalls, Mike A, Cornelis Blauwendraat, Costanza L Vallerga, Karl Heilbron, Sara Bandres-Ciga, Diana Chang, Manuela Tan, Demis A Kia, Alastair J Noyce, and Angli Xue. 2019. “Identification of Novel Risk Loci, Causal Insights, and Heritable Risk for Parkinson’s Disease: A Meta-Analysis of Genome-Wide Association Studies.” *The Lancet Neurology* 18 (12): 1091–1102.

Neale Lab. n.d. “Neale Lab GWAS Analysis of the UK Biobank.” http://www.nealelab.is/uk-biobank.

Nigg, Joel T, Alexis L Elmore, Neil Natarajan, Karen H Friderici, and Molly A Nikolas. 2016. “Variation in an Iron Metabolism Gene Moderates the Association Between Blood Lead Levels and Attention-Deficit/Hyperactivity Disorder in Children.” *Psychological Science* 27 (2): 257–69. https://doi.org/10.1177/0956797615618365.

Nikpay, Majid, Anuj Goel, Hong-Hee Won, Leanne M Hall, Christina Willenborg, Stavroula Kanoni, Danish Saleheen, Theodosios Kyriakou, Christopher P Nelson, and Jemma C Hopewell. 2015. “A Comprehensive 1000 Genomes-Based Genome-Wide Association Meta-Analysis of Coronary Artery Disease.” *Nature Genetics* 47 (10): 1121.

Okada, Yukinori, Di Wu, Gosia Trynka, Towfique Raj, Chikashi Terao, Katsunori Ikari, Yuta Kochi, Koichiro Ohmura, Akari Suzuki, and Shinji Yoshida. 2014. “Genetics of Rheumatoid Arthritis Contributes to Biology and Drug Discovery.” *Nature* 506 (7488): 376–81.

Orri, Massimiliano, Jean-Baptiste Pingault, Gustavo Turecki, Anne-Monique Nuyt, Richard E Tremblay, Sylvana M Côté, and Marie-Claude Geoffroy. 2021. “Contribution of Birth Weight to Mental Health, Cognitive and Socioeconomic Outcomes: Two-Sample Mendelian Randomisation.” *The British Journal of Psychiatry* 219 (3): 507–14. https://doi.org/DOI: 10.1192/bjp.2021.15.

Pagoni, Panagiota, Christina Dardani, Beate Leppert, Roxanna Korologou-Linden, George Davey Smith, Laura D Howe, Emma L Anderson, and Evie Stergiakouli. 2020. “Exploring the Causal Effects of Genetic Liability to ADHD and Autism on Alzheimer’s Disease.” *BioRxiv*, January, 2020.04.15.043380. https://doi.org/10.1101/2020.04.15.043380.

Pasman, Joëlle A, Karin J H Verweij, Zachary Gerring, Sven Stringer, Sandra Sanchez-Roige, Jorien L Treur, Abdel Abdellaoui, Michel G Nivard, Bart M L Baselmans, and Jue-Sheng Ong. 2018. “GWAS of Lifetime Cannabis Use Reveals New Risk Loci, Genetic Overlap with Psychiatric Traits, and a Causal Effect of Schizophrenia Liability.” *Nature Neuroscience* 21 (9): 1161–70.

Paternoster, Lavinia, Marie Standl, Johannes Waage, Hansjörg Baurecht, Melanie Hotze, David P Strachan, John A Curtin, Klaus Bønnelykke, Chao Tian, and Atsushi Takahashi. 2015. “Multi-Ethnic Genome-Wide Association Study of 21,000 Cases and 95,000 Controls Identifies New Risk Loci for Atopic Dermatitis.” *Nature Genetics* 47 (12): 1449.

Peng, Peng, Zirong Chen, Xiaolin Zhang, Zhongyin Guo, Fangyong Dong, Yu Xu, Yue He, Dongsheng Guo, and Feng Wan. 2021. “Investigating Causal Relationships Between Psychiatric Traits and Intracranial Aneurysms: A Bi-Directional Two-Sample Mendelian Randomization Study .” *Frontiers in Genetics* . https://www.frontiersin.org/article/10.3389/fgene.2021.741429.

Peyre, Hugo, Tabea Schoeler, Chaoyu Liu, Camille Michèle Williams, Nicolas Hoertel, Alexandra Havdahl, and Jean-Baptiste Pingault. 2021. “Combining Multivariate Genomic Approaches to Elucidate the Comorbidity between Autism Spectrum Disorder and Attention Deficit Hyperactivity Disorder.” *Journal of Child Psychology and Psychiatry* 62 (11): 1285–96. https://doi.org/https://doi.org/10.1111/jcpp.13479.

Pilling, Luke C, Chia-Ling Kuo, Kamil Sicinski, Jone Tamosauskaite, George A Kuchel, Lorna W Harries, Pamela Herd, Robert Wallace, Luigi Ferrucci, and David Melzer. 2017. “Human Longevity: 25 Genetic Loci Associated in 389,166 UK Biobank Participants.” *Aging (Albany NY)* 9 (12): 2504.

Plana-Ripoll, Oleguer, Carsten Bøcker Pedersen, Yan Holtz, Michael E. Benros, Søren Dalsgaard, Peter De Jonge, Chun Chieh Fan, et al. 2019. “Exploring Comorbidity Within Mental Disorders among a Danish National Population.” *JAMA Psychiatry* 76 (3): 259–70. https://doi.org/10.1001/jamapsychiatry.2018.3658.

Pulit, Sara L, Charli Stoneman, Andrew P Morris, Andrew R Wood, Craig A Glastonbury, Jessica Tyrrell, Loïc Yengo, Teresa Ferreira, Eirini Marouli, and Yingjie Ji. 2019. “Meta-Analysis of Genome-Wide Association Studies for Body Fat Distribution in 694 649 Individuals of European Ancestry.” *Human Molecular Genetics* 28 (1): 166–74.

Rao, S, A Baranova, Y Yao, J Wang, and F Zhang. 2022. “Genetic Relationships between Attention-Deficit/Hyperactivity Disorder, Autism Spectrum Disorder, and Intelligence.” *Neuropsychobiology*. https://doi.org/10.1159/000525411.

Riglin, Lucy, Beate Leppert, Christina Dardani, Ajay K Thapar, Frances Rice, Michael C O’Donovan, George Davey Smith, Evie Stergiakouli, Kate Tilling, and Anita Thapar. 2021. “ADHD and Depression: Investigating a Causal Explanation.” *Psychological Medicine* 51 (11): 1890–97. https://doi.org/DOI: 10.1017/S0033291720000665.

Sanchez-Roige, Sandra, Abraham A Palmer, Pierre Fontanillas, Sarah L Elson, Mark J Adams, David M Howard, Howard J Edenberg, et al. 2018. “Genome-Wide Association Study Meta-Analysis of the Alcohol Use Disorders Identification Test (AUDIT) in Two Population-Based Cohorts.” *American Journal of Psychiatry* 176 (2): 107–18. https://doi.org/10.1176/appi.ajp.2018.18040369.

Savage, Jeanne E, Philip R Jansen, Sven Stringer, Kyoko Watanabe, Julien Bryois, Christiaan A de Leeuw, Mats Nagel, et al. 2018. “Genome-Wide Association Meta-Analysis in 269,867 Individuals Identifies New Genetic and Functional Links to Intelligence.” *Nature Genetics* 50 (7): 912–19. https://doi.org/10.1038/s41588-018-0152-6.

Scott, Robert A, Laura J Scott, Reedik Mägi, Letizia Marullo, Kyle J Gaulton, Marika Kaakinen, Natalia Pervjakova, Tune H Pers, Andrew D Johnson, and John D Eicher. 2017. “An Expanded Genome-Wide Association Study of Type 2 Diabetes in Europeans.” *Diabetes* 66 (11): 2888–2902.

Shungin, Dmitry, Thomas W Winkler, Damien C Croteau-Chonka, Teresa Ferreira, Adam E Locke, Reedik Mägi, Rona J Strawbridge, Tune H Pers, Krista Fischer, and Anne E Justice. 2015. “New Genetic Loci Link Adipose and Insulin Biology to Body Fat Distribution.” *Nature* 518 (7538): 187–96.

Simón-Sánchez, Javier, Claudia Schulte, Jose M Bras, Manu Sharma, J Raphael Gibbs, Daniela Berg, Coro Paisan-Ruiz, et al. 2009. “Genome-Wide Association Study Reveals Genetic Risk Underlying Parkinson’s Disease.” *Nature Genetics* 41 (12): 1308–12. https://doi.org/10.1038/ng.487.

Soler Artigas, María, Cristina Sánchez-Mora, Paula Rovira, Vanesa Richarte, Iris Garcia-Martínez, Mireia Pagerols, Ditte Demontis, et al. 2020. “Attention-Deficit/Hyperactivity Disorder and Lifetime Cannabis Use: Genetic Overlap and Causality.” *Molecular Psychiatry* 25 (10): 2493–2503. https://doi.org/10.1038/s41380-018-0339-3.

Soler Artigas, María, Cristina Sánchez-Mora, Paula Rovira, Laura Vilar-Ribó, Josep Antoni Ramos-Quiroga, and Marta Ribasés. 2022. “Mendelian Randomization Analysis for Attention Deficit/Hyperactivity Disorder: Studying a Broad Range of Exposures and Outcomes.” *International Journal of Epidemiology*, June, dyac128. https://doi.org/10.1093/ije/dyac128.

Stringer, S, C C Minică, K J H Verweij, H Mbarek, M Bernard, J Derringer, K R van Eijk, et al. 2016. “Genome-Wide Association Study of Lifetime Cannabis Use Based on a Large Meta-Analytic Sample of 32 330 Subjects from the International Cannabis Consortium.” *Translational Psychiatry* 6 (3): e769–e769. https://doi.org/10.1038/tp.2016.36.

Sun, Benjamin B, Joseph C Maranville, James E Peters, David Stacey, James R Staley, James Blackshaw, Stephen Burgess, Tao Jiang, Ellie Paige, and Praveen Surendran. 2018. “Genomic Atlas of the Human Plasma Proteome.” *Nature* 558 (7708): 73–79.

Sun, Xiaohui, Bin Liu, Sitong Liu, David J H Wu, Jianming Wang, Yi Qian, Ding Ye, and Yingying Mao. 2022. “Sleep Disturbance and Psychiatric Disorders: A Bidirectional Mendelian Randomisation Study.” *Epidemiology and Psychiatric Sciences* 31: e26. https://doi.org/DOI: 10.1017/S2045796021000810.

Taylor, Amy E., Hannah J. Jones, Hannah Sallis, Jack Euesden, Evie Stergiakouli, Neil M. Davies, Stanley Zammit, et al. 2018. “Exploring the Association of Genetic Factors with Participation in the Avon Longitudinal Study of Parents and Children.” *International Journal of Epidemiology* 47 (4): 1207–16. https://doi.org/10.1093/ije/dyy060.

Tobacco, The, and Genetics Consortium. 2010. “Genome-Wide Meta-Analyses Identify Multiple Loci Associated with Smoking Behavior.” *Nature Genetics* 42 (5): 441.

Treur, Jorien L, Ditte Demontis, George Davey Smith, Hannah Sallis, Tom G Richardson, Reinout W Wiers, Anders D Børglum, Karin J H Verweij, and Marcus R Munafò. 2021. “Investigating Causality between Liability to ADHD and Substance Use, and Liability to Substance Use and ADHD Risk, Using Mendelian Randomization.” *Addiction Biology* 26 (1): e12849. https://doi.org/https://doi.org/10.1111/adb.12849.

Vilar-Ribó, Laura, Cristina Sánchez-Mora, Paula Rovira, Vanesa Richarte, Montserrat Corrales, Christian Fadeuilhe, Lorena Arribas, et al. 2021. “Genetic Overlap and Causality between Substance Use Disorder and Attention-Deficit and Hyperactivity Disorder.” *American Journal of Medical Genetics Part B: Neuropsychiatric Genetics* 186 (3): 140–50. https://doi.org/https://doi.org/10.1002/ajmg.b.32827.

Vink, Jacqueline M, Jorien L Treur, Joëlle A Pasman, and Arnt Schellekens. 2021. “Investigating Genetic Correlation and Causality between Nicotine Dependence and ADHD in a Broader Psychiatric Context.” *American Journal of Medical Genetics Part B: Neuropsychiatric Genetics* 186 (7): 423–29. https://doi.org/https://doi.org/10.1002/ajmg.b.32822.

Walters, Raymond K, Renato Polimanti, Emma C Johnson, Jeanette N McClintick, Mark J Adams, Amy E Adkins, Fazil Aliev, Silviu-Alin Bacanu, Anthony Batzler, and Sarah Bertelsen. 2018. “Transancestral GWAS of Alcohol Dependence Reveals Common Genetic Underpinnings with Psychiatric Disorders.” *Nature Neuroscience* 21 (12): 1656–69.

Wang, Heming, Jacqueline M Lane, Samuel E Jones, Hassan S Dashti, Hanna M Ollila, Andrew R Wood, Vincent T Van Hees, Ben Brumpton, Bendik S Winsvold, and Katri Kantojärvi. 2019. “Genome-Wide Association Analysis of Self-Reported Daytime Sleepiness Identifies 42 Loci That Suggest Biological Subtypes.” *Nature Communications* 10 (1): 1–12.

Wang, Kai, Lin Ding, Can Yang, Xingjie Hao, and Chaolong Wang. 2020. “Exploring the Relationship Between Psychiatric Traits and the Risk of Mouth Ulcers Using Bi-Directional Mendelian Randomization .” *Frontiers in Genetics* . https://www.frontiersin.org/article/10.3389/fgene.2020.608630.

Wang, Yufei, James D McKay, Thorunn Rafnar, Zhaoming Wang, Maria N Timofeeva, Peter Broderick, Xuchen Zong, Marina Laplana, Yongyue Wei, and Younghun Han. 2014. “Rare Variants of Large Effect in BRCA2 and CHEK2 Affect Risk of Lung Cancer.” *Nature Genetics* 46 (7): 736–41.

Warrier, Varun, Alex S.F. Kwong, Mannan Luo, Shareefa Dalvie, Jazz Croft, Hannah M. Sallis, Jessie Baldwin, et al. 2021. “Gene–Environment Correlations and Causal Effects of Childhood Maltreatment on Physical and Mental Health: A Genetically Informed Approach.” *The Lancet Psychiatry* 8 (5): 373–86. https://doi.org/10.1016/S2215-0366(20)30569-1.

Warrington, Nicole M, Robin N Beaumont, Momoko Horikoshi, Felix R Day, Øyvind Helgeland, Charles Laurin, Jonas Bacelis, et al. 2019. “Maternal and Fetal Genetic Effects on Birth Weight and Their Relevance to Cardio-Metabolic Risk Factors.” *Nature Genetics* 51 (5): 804–14. https://doi.org/10.1038/s41588-019-0403-1.

Wendt, Frank R, Carolina Muniz Carvalho, Gita A Pathak, Joel Gelernter, and Renato Polimanti. 2019. “Deciphering the Biological Mechanisms Underlying the Genome-Wide Associations between Computerized Device Use and Psychiatric Disorders.” *Journal of Clinical Medicine* . https://doi.org/10.3390/jcm8122040.

Wood, Andrew R, Tonu Esko, Jian Yang, Sailaja Vedantam, Tune H Pers, Stefan Gustafsson, Audrey Y Chu, Karol Estrada, Jian’an Luan, and Zoltán Kutalik. 2014. “Defining the Role of Common Variation in the Genomic and Biological Architecture of Adult Human Height.” *Nature Genetics* 46 (11): 1173–86.

Wootton, Robyn E, Rebecca C Richmond, Bobby G Stuijfzand, Rebecca B Lawn, Hannah M Sallis, Gemma M J Taylor, Gibran Hemani, et al. 2020. “Evidence for Causal Effects of Lifetime Smoking on Risk for Depression and Schizophrenia: A Mendelian Randomisation Study.” *Psychological Medicine* 50 (14): 2435–43. https://doi.org/DOI: 10.1017/S0033291719002678.

Wray, N R, S Ripke, M Mattheisen, M Trzaskowski, E M Byrne, A Abdellaoui, M J Adams, et al. 2018. “Genome-Wide Association Analyses Identify 44 Risk Variants and Refine the Genetic Architecture of Major Depression.” *Nat Genet* 50 (5): 668–81. https://doi.org/10.1038/s41588-018-0090-3.

Wray, Naomi R., Sang Hong Lee, Divya Mehta, Anna A.E. Vinkhuyzen, Frank Dudbridge, and Christel M. Middeldorp. 2014. “Research Review: Polygenic Methods and Their Application to Psychiatric Traits.” *Journal of Child Psychology and Psychiatry and Allied Disciplines* 55 (10): 1068–87. https://doi.org/10.1111/jcpp.12295.

Yang, Jian, Xiaoyan He, Li Qian, Binbin Zhao, Yajuan Fan, Fengjie Gao, Bin Yan, Feng Zhu, and Xiancang Ma. 2022. “Association between Plasma Proteome and Childhood Neurodevelopmental Disorders: A Two-Sample Mendelian Randomization Analysis.” *EBioMedicine* 78: 103948.

Yang, Jian, Bin Yan, Binbin Zhao, Yajuan Fan, Xiaoyan He, Lihong Yang, Qingyan Ma, et al. 2020. “Assessing the Causal Effects of Human Serum Metabolites on 5 Major Psychiatric Disorders.” *Schizophrenia Bulletin* 46 (4): 804–13. https://doi.org/10.1093/schbul/sbz138.

Yao, Yao, Chun’e Li, Peilin Meng, Bolun Cheng, Shiqiang Cheng, Li Liu, Xuena Yang, Yumeng Jia, Yan Wen, and Feng Zhang. 2022. “An Atlas of Genetic Correlations between Gestational Age and Common Psychiatric Disorders.” *Autism Research* 15 (6): 1008–17. https://doi.org/https://doi.org/10.1002/aur.2719.

Yengo, Loic, Julia Sidorenko, Kathryn E Kemper, Zhili Zheng, Andrew R Wood, Michael N Weedon, Timothy M Frayling, Joel Hirschhorn, Jian Yang, and Peter M Visscher. 2018. “Meta-Analysis of Genome-Wide Association Studies for Height and Body Mass Index In∼ 700000 Individuals of European Ancestry.” *Human Molecular Genetics* 27 (20): 3641–49.

Zhang, Fuquan, Ancha Baranova, Chao Zhou, Hongbao Cao, Jiu Chen, Xiangrong Zhang, and Mingqing Xu. 2021. “Causal Influences of Neuroticism on Mental Health and Cardiovascular Disease.” *Human Genetics* 140 (9): 1267–81. https://doi.org/10.1007/s00439-021-02288-x.

Zhao, Sizheng Steven, Yu Qian, Sarah L Mackie, Chengping Wen, and Yingying Mao. 2021. “Genetically Predicted Serum Urate Levels Have No Causal Role on Depression or Other Psychiatric Disorders.” *Clinical Rheumatology* 40 (9): 3729–33. https://doi.org/10.1007/s10067-021-05718-3.

Zhu, Zhaozhong, Xi Zhu, Cong-Lin Liu, Huwenbo Shi, Sipeng Shen, Yunqi Yang, Kohei Hasegawa, Carlos A Camargo, and Liming Liang. 2019. “Shared Genetics of Asthma and Mental Health Disorders: A Large-Scale Genome-Wide Cross-Trait Analysis.” *European Respiratory Journal* 54 (6): 1901507. https://doi.org/10.1183/13993003.01507-2019.

Zhu, Zhihong, Futao Zhang, Han Hu, Andrew Bakshi, Matthew R Robinson, Joseph E Powell, Grant W Montgomery, Michael E Goddard, Naomi R Wray, and Peter M Visscher. 2016. “Integration of Summary Data from GWAS and EQTL Studies Predicts Complex Trait Gene Targets.” *Nature Genetics* 48 (5): 481–87.
